# Comprehensive study of mtDNA among Southwest Asian dogs contradicts independent domestication of wolf, but implies dog–wolf hybridization

**DOI:** 10.1002/ece3.35

**Published:** 2011-11

**Authors:** Arman Ardalan, Cornelya F C Kluetsch, Ai-bing Zhang, Metin Erdogan, Mathias Uhlén, Massoud Houshmand, Cafer Tepeli, Seyed Reza Miraei Ashtiani, Peter Savolainen

**Affiliations:** 1 Department of Gene Technology, KTH–Royal Institute of Technology, Science for Life Laboratory171 21 Solna, Sweden; 2 Department of Medical Biology and Genetics, Afyon Kocatepe University03200 Afyonkarahisar, Turkey; 3 Department of Proteomics, KTH - Royal Institute of Technology106 91 Stockholm, Sweden; 4 Department of Medical Biotechnology, National Institute of Genetic Engineering and Biotechnology (NIGEB)14965/161 Tehran, Iran; 5 Department of Animal Science, Selcuk University42031 Konya, Turkey; 6 Department of Animal Science, University of Tehran4111 Karaj, Iran

**Keywords:** *Canis familiaris*, domestication, fertile crescent, hybridization, mitochondrial DNA

## Abstract

Studies of mitochondrial DNA (mtDNA) diversity indicate explicitly that dogs were domesticated, probably exclusively, in southern East Asia. However, Southwest Asia (SwAsia) has had poor representation and geographical coverage in these studies. Other studies based on archaeological and genome-wide SNP data have suggested an origin of dogs in SwAsia. Hence, it has been suspected that mtDNA evidence for this scenario may have remained undetected. In the first comprehensive investigation of genetic diversity among SwAsian dogs, we analyzed 582 bp of mtDNA for 345 indigenous dogs from across SwAsia, and compared with 1556 dogs across the Old World. We show that 97.4% of SwAsian dogs carry haplotypes belonging to a universal mtDNA gene pool, but that only a subset of this pool, five of the 10 principal haplogroups, is represented in SwAsia. A high frequency of haplogroup B, potentially signifying a local origin, was not paralleled with the high genetic diversity expected for a center of origin. Meanwhile, 2.6% of the SwAsian dogs carried the rare non-universal haplogroup d2. Thus, mtDNA data give no indication that dogs originated in SwAsia through independent domestication of wolf, but dog–wolf hybridization may have formed the local haplogroup d2 within this region. Southern East Asia remains the only region with virtually full extent of genetic variation, strongly indicating it to be the primary and probably sole center of wolf domestication. An origin of dogs in southern East Asia may have been overlooked by other studies due to a substantial lack of samples from this region.

## Introduction

**Figure 1:**
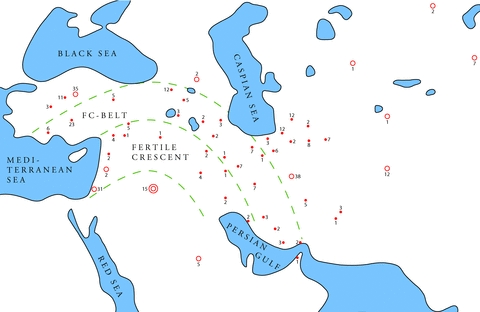
Map of SwAsia showing FC and FC-belt, together forming FC-xtd, with sampling locations and number of samples collected in each location. Bullet point = exact location; circle = approximate area; double circle = general SwAsia.

Southwest Asia (SwAsia) was probably the first of only a handful of regions, together with China and Mesoamerica, in which independent domestication of plants and animals led to the transition from hunting and gathering to sedentary farming, and a subsequent development of the first complex societies (Colledge et al. 2004; Jobling et al. 2004; Zeder 2008). In SwAsia, large-scale farming developed within the so-called Fertile Crescent (FC: a crescent-shaped region stretching from the Persian Gulf, along western Zagros Mountains and southern Taurus Mountains, to the eastern Mediterranean coast, confined from south by the Syrian Desert; see Fig. 1). However, the actual domestication and the earliest development of domesticates, particularly of animals, took place to a large extent also in the surrounding mountainous area along the north and east of the FC (here called FC-belt:; see Fig. 1) (Diamond 2002; Zeder 2008). The process of domestication started at least 11,000 before present (BP) in SwAsia (Zeder, 2008), preceding that in China (≈10,000 BP [Lu et al. 2009]) and Mesoamerica (≈6000 BP [MacNeish and Eubanks 2000]).

The domestic dog (*Canis familiaris*) was probably the earliest domesticated animal. Archaeological evidence from SwAsia indicates that domestic dogs existed by 11,500 years BP (Dayan 1994; Raisor 2005). Thus, dogs possibly uniquely originated in the Mesolithic among hunter gatherers, before all other domesticates, which developed together with or after the emergence of farming. Still earlier dates for the presence of domestic dog have been reported from Europe (Sablin and Khlopachev 2002; Street 2002), but the evidence does not seem conclusive (Wang et al. 2008; Napierala and Uerpmann 2010). Due to difficulties in distinguishing wild canids from dogs, and also geographical bias in the extent of detailed analysis of ancient animal materials, interpretation of the archaeological record is problematic (Underhill 1997; Raisor 2005; Wang et al. 2008).

Importantly, the archaeological sites with the earliest reasonably firm evidence of dogs are situated within the FC, in connection with the Natufian, a culture of sedentary hunter-gatherers directly preceding the Neolithic and the emergence of farming (Dayan 1994). Dogs are also frequently depicted in art at early times in different parts of SwAsia (Clutton–Brock 1981; Przezdziecki et al. 2001). Therefore, one of the most prominent theories about the geographical origin of the domestic dog has been that they originated in SwAsia, supposedly in the FC (Clutton–Brock 1981).

However, studies of mitochondrial DNA (mtDNA) instead strongly indicate a single origin of dogs in East Asia (Savolainen et al. 2002), particularly in the southern part, in a region dubbed Asia South of Yangtze River (ASY) (Pang et al. 2009). This is based on homogenous sharing of a common gene pool among all populations across the Old World, but the full extent of the diversity being present only in ASY. The mtDNA data also indicate that the dogs originated less than 16,300 years ago from at least 51 female wolves.

The study of mtDNA by Pang et al. (2009) is the only globally comprehensive study of the genetic variation in dogs so far, with a sample of 1542 dogs from across the Old World, including 133 from SwAsia. Based on 582 bp of the mtDNA Control Region, the dog mtDNA haplotypes were distributed in six phylogenetic groups, called haplogroups A to F, which are dispersed in different parts of the global distribution of wolf haplotypes (Pang et al. 2009). The three major dog haplogroups, A, B, and C, were carried by almost 100% of the dogs around the world, and represented in virtually every dog population of the Old World at markedly similar proportions (A: 55–85%, B: 10–35%, C: 5–15%). Furthermore, 14 haplotypes termed Universal Types (UTs) were shared by ≈80% of all individuals in Europe, SwAsia, and East Asia, and an additional 15% carried haplotypes differing by a single substitution from a UT, so that ≈95% carried what was termed as UT-derived haplotype (UTd, to include both the derived haplotypes and the UTs). Thus, the dogs in the Old World, including SwAsia, carry almost exclusively haplotypes that are shared in a homogenous gene pool composed of identical or nearly identical haplotypes. However, in ASY only 40.5% of the dogs had a UT and 52.5% a UTd; instead, almost 50% carried haplotypes that, while belonging to the universal haplogroups A, B, and C, were unique and phylogenetically distinct. Analysis of complete mtDNA genomes revealed haplogroups A, B, and C to be composed of 10 distinct sub-haplogroups in total (six, two, and two, respectively, in haplogroups A, B, and C). All 10 sub-haplogroups were represented in ASY, while other regions had only a subset of four (e.g., in Europe) to seven (e.g., in other East Asian regions) haplogroups, explaining the difference in diversity for the 582-bp segment between ASY and other regions. SwAsia had five sub-haplogroups (only one of the six sub-haplogroups in haplogroup A, and two in each of haplogroups B and C); it, therefore, seems unlikely that the universal gene pool originated in SwAsia.

The universal sharing at similar proportions of the three haplogroups, A, B, and C, strongly indicates a single geographical origin for all dogs. It is unlikely that geographically separate origins of dogs would result in dog populations carrying the same haplogroups, since wolf populations in different parts of the world carry different haplotypes (Pang et al. 2009). Furthermore, had the three haplogroups had separate origins from different wolf populations (carrying different haplogroups), the locally obtained haplogroup would have been carried by initially 100% of the dogs in each center of origin, and would normally have remained at majority until today; population genetic simulations (Pang et al. 2009) show that very extreme migration rates would have been demanded for today's homogenous sharing of the three haplogroups to form. Therefore, the mtDNA data clearly indicate a single origin for the universal mtDNA gene pool, and since ASY is the only region with full coverage of haplogroups A, B, and C, it is the most probable region to have housed a simultaneous origin of the three haplogroups.

A direct comparison of the global populations of dog and wolf may seem a straightforward approach for deducing the origins of dogs. However, available wolf data are restricted and the wolf has become extinct in large parts of the world, for example, ASY, preventing a comprehensive comparison. Nevertheless, the available phylogeny data (Pang et al. 2009) agree with an origin of dogs from East Asia; dog mtDNA haplogroup A contains haplotypes from North Chinese and Mongolian wolves and haplogroup B contains haplotypes from North Chinese, European, as well as SwAsian wolves, while haplogroups C, D, E, and F are distant from wolf haplotypes.

**Figure 2:**
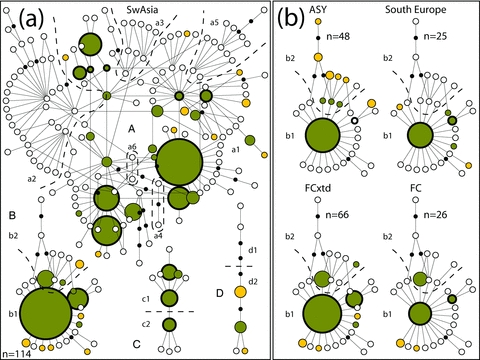
MS networks showing regional distribution and genetic relationships among haplotypes. Circles represent haplotypes, and black bullets represent assumed intermediary haplotypes. Lines represent one substitutional mutation. The size of the circles is proportional to the frequency of the haplotype. The UTs are indicated by bold lines. Haplotype A5 was for the first time found among SwAsian dogs, and since it had earlier been found in Europe and East Asia, it was designated as a new, the 15th, UT (see Fig. S2 for the exact location of the haplotypes). The sub-haplogroups, identified by analysis of complete mtDNA genomes (Pang et al. 2009), are separated by dashed lines. Green color indicates haplotypes shared with other regions, while orange color indicates unique haplotypes of the region: (A) representation of the total sample for SwAsia; (B) representation of haplotypes in haplogroup B for ASY, South Europe, FC-xtd, and FC.

In addition to the major haplogroups, A, B, and C, also haplogroup D (specifically sub-haplogroup d2) was found in SwAsia, in 2.3% of the dogs (Fig. 2A). Haplogroup D is the only haplogroup exclusively found outside East Asia and therefore probably originated elsewhere, separate from haplogroups A, B, and C (Pang et al. 2009; Klütsch et al. 2011). sub-haplogroup d2 is geographically restricted to SwAsia and the Mediterranean region, and possibly originated in SwAsia, but the low frequency indicates an origin through dog–wolf crossbreeding, rather than independent domestication of wolf.

Thus, in contrast to inferences made from archaeology, mtDNA data have given no indication that dogs may have had an independent origin in SwAsia. However, the SwAsian dog population was not adequately sampled in Pang et al. (2009): the total number of samples (*n* = 133) was relatively small and, more importantly, different geographical regions within SwAsia were not comprehensively covered (e.g., FC, the historically most important part of SwAsia, was represented by only 17 samples). Furthermore, the samples were generally from dogs of specific breeds or types, particularly sighthound, not properly reflecting the general dog population. This raises the concern (Boyko et al. 2009) that the low mtDNA diversity compared to dogs from ASY (generally non-breed dogs from rural areas) may reflect a difference between breed dogs and “village dogs,” rather than between SwAsian and East Asian dogs.

The mtDNA diversity for SwAsian dogs largely followed the general pattern of the Old World populations, but it deviated by having distinctly higher frequency of haplogroup B (carried by 33% of all individuals) than all other regions (overall 15–25%), and by the presence of sub-haplogroup d2. A frequency of at least 50% as well as a distinguished diversity would be anticipated for domestication-derived haplogroups within their region of origin. However, the sparse and uneven sampling of SwAsian dogs implies a possibility that sub-regions with considerably higher frequencies of haplogroup B and sub-haplogroup d2, or even novel haplogroups, were not detected. Importantly, a study of genome-wide SNP variation among domestic dogs and wolves (vonHoldt et al. 2010) showed dogs to share more unique multilocus haplotypes with wolves from the Middle East than with wolves from Northern China, Europe, and America, therefore suggesting SwAsia as the major source of genetic diversity for the domestic dog. However, the fact that wolf samples from ASY were totally lacking implies that if dogs originated in ASY it could not have been detected by this study. Nevertheless, the analysis by vonHoldt et al. (2010) calls for a more comprehensive sample from SwAsia to be analyzed for mtDNA, to ensure that evidence for an independent domestication has not been omitted.

**Figure 3:**
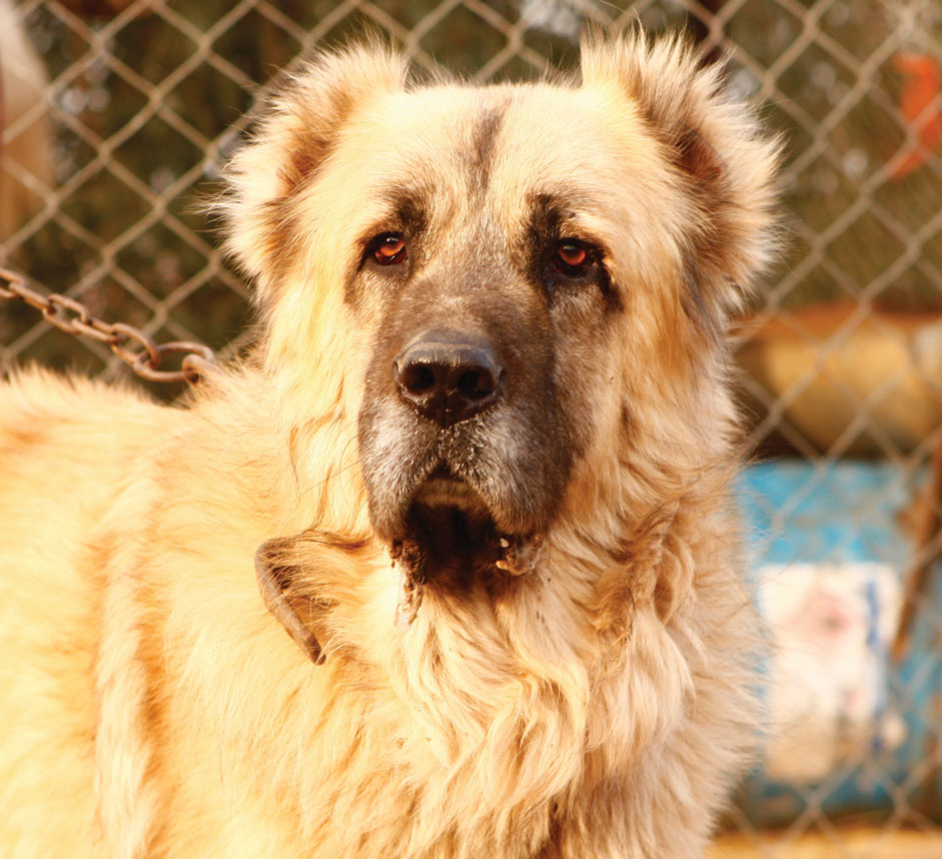
Guard dog of long-haired Sarabi ecotype with cropped ears, northwest Iran. Photo by Ali Golshan.

Regardless of where the dog originated, SwAsia holds a central place in the history of dogs. With its geographical position and historical role as a bridge between East and West, SwAsia provides a basis for understanding the origin of the European and African dog populations. SwAsia was probably also important in the development of dog phenotypes. The morphologically distinct sighthound is an indigenous dog type across the region (in much of SwAsia known collectively as “Tazi,” meaning “the runner/raider” in Persian language). Sighthounds are shown to cluster distantly from other types of dogs (Parker et al. 2004; vonHoldt et al. 2010), and with a local history of at least 5500 years as inferred from ancient art and other archaeological evidence (Przezdziecki et al. 2001), are commonly suggested to have evolved in SwAsia (American Kennel Club 2006). However, working dogs of several mastiff ecotypes such as Akbash and Kangal (mainly from Anatolia) and Kordi, Sarabi (Fig. 3), and Qahderijani (mainly from the Persian Plateau), and also spitz-type pariah and hunting dogs, are most typical to the SwAsian region. The companionship of the dog was long practiced in ancient SwAsia (Clutton–Brock 1999; Przezdziecki et al. 2001); and under the influence of Zoroastrian faith and mythology, the dog gained a spiritual position (Afshar 1990; Simoons 1994), that survived also into Roman Mithraism (a cult closely linked to Zoroastrianism) (Smart 1996). Ritual burials of dogs have been unearthed, like elsewhere, in SwAsia (Wapnish and Hesse 1993), where the consumption of dog flesh has not been evident (Simoons 1994; Olsen 2000) unlike many other regions: among others Southeast Asia, where the practice has continued to modern day (Simoons 1991, 1994).

In order to study whether, and to what extent, independent dog domestication or dog–wolf hybridization may have occurred in SwAsia, we here perform the first comprehensive analysis of mtDNA diversity, based on extensive samples of indigenous dogs from the Persian Plateau, Anatolia, and the FC with related areas (i.e., FC with FC-belt, together called here FC-xtd: FC-extended; see Fig. 1). We specifically examine the possibility that sub-regions within SwAsia have considerably higher frequencies of haplogroup B and sub-haplogroup d2 than previously shown, or still undetected haplogroups, to ascertain whether haplogroup B could have had a separate origin in SwAsia rather than together with haplogroups A and C in ASY, and whether sub-haplogroup d2 is a result of wolf domestication, or of dog–wolf crossbreeding.

## Materials and Methods

### Samples

We studied 345 dogs from across large parts of SwAsia, and 1556 dogs covering a large range of populations from all over the world (Fig. 1; Tables 1, S1, and S2), for mtDNA diversity by DNA sequencing of 582 bp of the Control Region (nucleotide positions 15.458–16.039 of the mitochondrial genome). DNA sequencing was carried out for 325 new samples (212 of which from SwAsia and 107 from Europe) and 1576 DNA sequences were retrieved from literature (Pang et al. 2009). The SwAsian sample included 151 dogs from the FC-xtd, and extensive samples from the Persian Plateau (sampled in Iran and Afghanistan; *n* = 169) and Anatolia (sampled in Turkey; *n* = 111). To represent the indigenous dog population across SwAsia, the samples were collected from a large number of different locations mostly situated in rural areas (Fig. 1). Following the distribution of the dog population in the region, our sample set mainly included working dogs of no specific breed, but predominantly of different mastiff ecotypes (Table S1), and also populations of the indigenous sighthounds, stray dogs, and Canaan dogs (a stock recently developed from Bedouin pariah dogs of the Levant [Levine 2003]). Major influences from foreign breed dogs on the SwAsian dog population seem unlikely since such dogs are rare outside the upper social strata of urban areas. Historically, SwAsia has not experienced extensive import of dogs from other regions, and has not been under long-term foreign control in modern history (as in the case of, for example, Africa). Therefore, the sample collection from SwAsia is, to our knowledge, a faithful representation of the indigenous dog population. Importantly, since the samples from ASY, obtained from the literature (Pang et al. 2009), were also mainly from working dogs in rural areas, the comparisons of mtDNA diversity between SwAsia and ASY are performed based on very similar grounds.

**Table 1:** Detailed structure and diversity information for different regions. See Table S2 for definitions of the regions

Population	NABC (DEF)^1^	nA (%)^2^	nB (%)^2^	nC (%)^2^	nHT^3^	nHTuq (%)^4^	nHTrsp56^5^	HTdiv (SD)^6^	nsubHG^7^	%UT^8^	%UTd^9^
Total	1850 (51)	1342 (70.6)	368 (19.4)	140 (7.4)	206	-	28.75	0.944 (0.003)	10	63.7	79.4
SwAsia	336 (9)	200 (58.0)	114 (33.0)	22 (6.4)	49	17 (34.7)	20.59	0.893 (0.010)	5	77.7	94.5
Persian Plateau	164 (5)	106 (62.7)	46 (27.2)	12 (7.1)	29	8 (27.6)	18.25	0.900 (0.011)	5	80.5	92.3
Anatolia	107 (4)	64 (57.7)	35 (31.5)	8 (7.2)	28	5 (17.9)	18.84	0.881 (0.019)	4	76.6	95.5
Fertile Crescent	57 (0)	30 (52.6)	26 (45.6)	1 (1.7)	13	2 (15.4)	12.93	0.868 (0.024)	4	71.9	98.2
FC-belt^10^	92 (2)	43 (45.7)	40 (42.5)	9 (9.6)	29	5 (17.2)	22.25	0.885 (0.026)	5	77.7	93.6
FC-xtd^11^	149 (2)	73 (48.3)	66 (43.7)	10 (6.6)	33	7 (21.2)	20.28	0.885 (0.019)	5	75.5	95.4
SwAexclFC-xtd^12^	187 (7)	127 (65.5)	48 (24.7)	12 (6.2)	36	10 (27.8)	19.92	0.887 (0.014)	5	79.4	93.8
SwAexclSight^13^	281 (8)	181 (62.6)	79 (27.3)	21 (7.3)	41	13 (31.7)	20.24	0.907 (0.009)	5	78.9	94.5
SwAsianSight^14^	55 (1)	19 (33.9)	35 (62.5)	1 (1.8)	14	4(28.6)	14.00	0.643 (0.071)	4	71.4	94.6
Europe	433 (27)	302 (65.6)	95 (20.6)	36 (7.8)	51	19 (37.2)	20.92	0.927 (0.005)	4	76.7	92.6
NorthContErp^15^	141 (1)	90 (63.4)	30 (21.1)	21 (14.8)	22	3 (13.6)	15.57	0.916 (0.007)	4	91.5	97.9
South Europe	111 (6)	82 (70.1)	25 (21.4)	4 (3.4)	31	9 (29.0)	21.57	0.921 (0.012)	4	69.2	90.6
Africa	57 (1)	48 (82.8)	7 (12.1)	2 (3.4)	23	6 (26.1)	22.61	0.939 (0.014)	4	65.5	93.1
IndianSubcont^16^	62 (0)	49 (79.0)	5 (8.1)	8 (12.9)	23	6 (26.1)	21.96	0.930 (0.016)	5	54.8	80.6
Siberia	60 (2)	39 (62.9)	13 (21.0)	8(12.9)	22	8 (36.4)	21.39	0.947 (0.013)	4	50.0	72.6
Japan	118 (3)	76 (62.8)	24 (19.8)	18 (14.9)	28	8 (28.6)	20.94	0.941 (0.007)	7	58.7	82.6
Northern China	273 (0)	200 (73.3)	52 (19.0)	21 (7.7)	44	16 (36.4)	21.37	0.924 (0.007)	7	71.4	82.8
ASY^17^	339 (2)	273 (80.1)	48 (14.1)	18 (5.3)	88	54 (61.4)	30.07	0.959 (0.004)	10	40.5	52.5
East Asia	855 (12)	658 (75.9)	133 (15.3)	64 (7.4)	131	101 (77.1)	28.90	0.952 (0.003)	10	54.9	68.6
West Eurasia	769 (36)	502 (62.4)	209 (26.0)	58 (7.2)	74	37 (50.0)	21.81	0.916 (0.005)	5	77.1	93.4

1 Total number of samples divided for the universal haplogroups A, B, and C (non-universal haplogroups D, E, and F).

2 Number of samples belonging to each of haplogroups A, B, and C (proportions of the total samples of the region).

3 Number of haplotypes.

4 Number of haplotypes unique to the region (proportions of the total haplotypes of the region).

5 Number of haplotypes obtained from resampling of size 56 (the size of the smallest sample) with 1000 replications.

6 Haplotype diversity (standard deviation).

7 Number of sub-haplogroups.

8 Proportion (%) of the samples with a UT.

9 Proportion (%) of the samples with a UTd.

10 Fertile Crescent-belt.

11 Fertile Crescent-extended.

12 Southwest Asia excluding FC-extended.

13 Southwest Asia excluding sighthounds.

14 Southwest Asian sighthounds.

15 North continental Europe.

16 Indian subcontinent.

17 Asia South of Yangtze.

### Sample collection

Samples were collected in the form of buccal cells or hairs. Buccal samples were collected by swabbing the inside of the cheek using sponge swabs, and applied to Whatman® FTA cards (Whatman International Ltd., Maidstone, UK) that were then stored at room temperature, according to the manufacturer's instructions. Hairs were plucked mostly around the belly or inside of thigh to obtain hairs carrying follicular epithelium. The samples were kept in nylon bags at room temperature until taken to laboratory and stored at 4°C for immediate use, or –20°C for longer storage.

### DNA extraction

The root portion (approximately 1 cm) of 5–10 hair strands for each individual was digested in a mixture of 10 mM tris-HCl, pH 8.0, 0.9% polyoxyethylene-10-laurylether (POLE), 50 µg/mL proteinase K, and 35 mM dithiothreitol (DTT), at 50°C overnight. The mixture was subjected to vortex mixing, once during and once at the end of incubation. A final heat treatment at 90°C for 10 min was performed to deactivate proteinase K for the following polymerase chain reaction (PCR) amplification. Sample tubes were thereafter centrifuged, and the supernatant was used for PCR. DNA from buccal epithelial cells was purified according to the Whatman® purification protocol.

### PCR amplification

DNA from hair samples was amplified in two separate PCR reactions: 742 bp of mtDNA Control Region was amplified in two overlapping fragments using the primer pairs H15360 (5′-ATT ACC TTG GTC TTG TAA ACC-3′) and L15784 (5′-CTG AAG TAA GAA CCA GAT GCC-3′), and H15693 (5′-AAT AAG GGC TTA ATC ACC ATG C-3′) and L16102 (5′-AAC TAT ATG TCC TGA AAC CAT TG-3′) (Savolainen et al. 2002). The amplifications were performed on a Primus 96 plus thermal cycler (MWG-Biotech, Herts, UK) in 50-µl reactions, containing roughly 20-ng template DNA, 20 mM each dNTP, 10 mM tris-HCl (pH 8.3), 25 mM KCl, 0.75 mM MgCl2, 10 pmol each primer, and 0.2 U Smar*Taq*™ DNA Polymerase (CinnaGen, Tehran, Iran). Thermal cycling consisted of initial denaturation at 95°C for 10 min, 40 cycles of 60-sec denaturation at 95°C, 40-sec primer annealing at 59°C, and 100-sec extension at 72°C, and a final extension at 72°C for 10 min.

For buccal epithelium samples, a nested primer configuration was used in order to improve specificity for the target sequence. Primers H15404 (5′-CCT AAG ACT CAA GGA AGA AGC-3′) and L16102 (5′-AAC TAT ATG TCC TGA AAC CAT TG-3′) were used for the outer reaction, and primers H15430 (5′-TCC ACC ATC AGC ACC CAA AG-3′) and L16092 (5′-CTG AAA CCA TTG ACT GAA TAG-3′) for the inner. Template DNA for the outer PCR reaction was added in the form of one FTA card disc to a 45-µl mixture consisting of 2 mM MgCl2, 20 mM Tris-HCl (pH 8.4), 50 mM KCl, 200 mM of each dNTP, 1 unit of Platinum® Taq DNA Polymerase.

### DNA Sequence analysis

For sequencing, the two inner primers from the nested PCR, H15430 (5′-TCC ACC ATC AGC ACC CAA AG-3′) and L16092 (5′-CTG AAA CCA TTG ACT GAA TAG-3′), and two additional primers H15706 (5′-CAC CAT GCC TCG AGA AAC CAT-3′) and L15791 (5′-ATG GCC CTG AAG TAA GAA CC-3′) were used. The Cycle sequencing reaction consisted of 17.5 µl-1× cycle sequencing buffer (26 mM Tris-HCl pH 9.0, 6.5 mM MgCl2), 1-µl ABI PRISM Big Dye Terminator Cycle Sequencing Ready Reaction Kit v3.1, 0.5-µl sequencing primer, and 1-µl amplification product for a total volume of 20 µl. The cycle sequencing program consisted of 35 cycles of denaturation at 96°C for 10 sec, primer annealing at 55°C for 15 sec, and extension at 60°C for 4 min, and the reaction was run in a Hybaid MBS 0.2S. The cycle sequencing products were ethanol precipitated and analyzed on an ABI 3700 DNA sequencer (Applied Biosystems, Foster City, CA, USA) according to the manufacturer's instructions. DNA sequences were edited in *Chromas 1.61* (Technelysium Pty Ltd., Helensville, Queensland, Australia) and *Sequencing Analysis 3.7* (Applied Biosystems) and assembled into contigs using *Seqman 5.00* (DNASTAR Inc., Madison, WI, USA) and *Sequencher 4.1* (Gene Codes Corporation, Ann Arbor, MI, USA). The consensus sequences were further aligned in *ClustalX 1.81* (Thompson et al. 1997) and *SeqEd* (Applied Biosystems).

### Phylogenetic and statistical analysis

Novel haplotypes were named in accordance with the nomenclature from Savolainen et al. (2002) and Pang et al. (2009) (Table S1). Minimum-spanning (MS) networks, showing the shortest genetic distance (in terms of the number of substitutions only) between the haplotypes, were inferred using the distance matrices generated in Arlequin ver. 3.5 (Excoffier and Lischer 2010), and drawn manually. Haplotype diversity (with standard deviation) was also estimated using Arlequin ver. 3.5. Population data including number of haplotypes, proportions of individuals carrying haplogroups A, B, and C, and frequency of individuals carrying UTs (15 haplotypes carried by dogs in Europe, SwAsia, as well as East Asia) and UTds (haplotypes which are either a UT or differ by one substitutional step from a UT) were calculated using an in-house developed computer program. For comparison of the number of haplotypes among populations, sample sizes were normalized by resampling without replacement (1000 replications) adjusted to the size of the smallest informative sample, using an in-house developed program.

## Results

We analyzed 582 bp of the mtDNA Control Region for 345 dogs from SwAsia, including 151 dogs from within or near the FC, and large samples from the Persian Plateau (*n* = 169) and Anatolia (*n* = 111), in the context of a collective sample of totally 1901 dogs from around the world (Figs. 1 and 2A; Tables 1 and S1). Among the 325 new samples sequenced in this study, 17 new haplotypes (based on substitutions only, 21 if indels are also taken into account as a source of variation) were identified: 11, 5, and 1 haplotypes from haplogroups A, B, and D, respectively (Table S3). Ten of these new haplotypes were found among SwAsian dogs (Fig. 2A), two in north continental Europe, and five in south Europe (Table S3). All of these haplotypes grouped with haplogroups previously reported for their corresponding regions (Pang et al. 2009). Therefore, SwAsian dogs had mtDNA haplotypes grouping within the universally occurring haplogroups A, B, and C (specifically within sub-haplogroups a1, newb1, newb2, c1, and c2) and within haplogroup D (sub-haplogroup d2) (Fig. 2A). Importantly, no entirely novel haplogroups were found in SwAsia, which implies that there were no haplogroups exclusive to this part of the world. As illustrated in the MS networks (Fig. 2A), large parts of the diversity for haplogroup A corresponding to sub-haplogroups a2–a6 remains unrepresented by the SwAsian dogs. The five sub-haplogroups a2–a6 are therefore almost exclusively restricted to East Asia, and sub-haplogroups a3, a5, and a6 to ASY (Fig. 3; Table 1). This implies that the only region with full diversity of mtDNA (all 10 sub-haplogroups) is ASY. Other measures of diversity, for example, number of haplotypes (adjusted for sample size) and unique haplotypes, and haplotype diversity had values similar to those previously reported (Pang et al. 2009). Thus, the values for SwAsia were comparable to those of the other regions across the Old World except for ASY, which stands out with exceptionally high values (Table 1).

**Figure 4:**
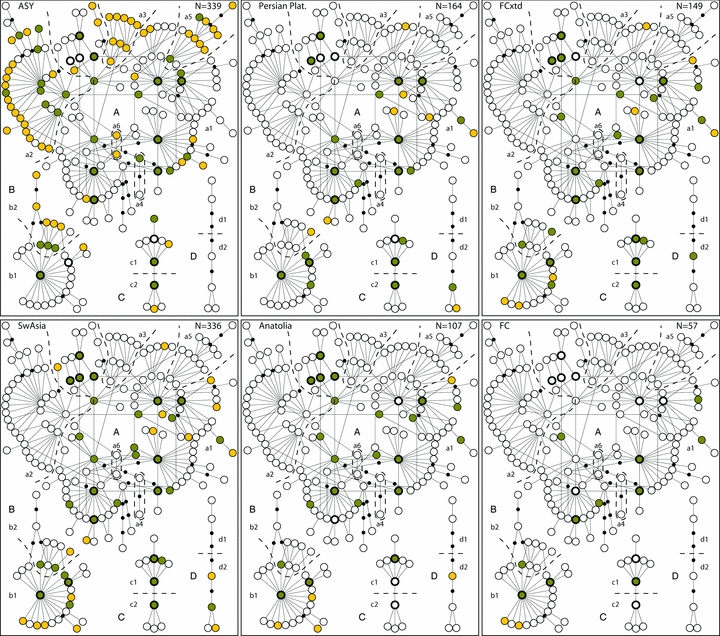
MS networks showing representation (but not frequency) for ASY, SwAsia, the Persian Plateau, Anatolia, FC-xtd, and FC. See Figure 2 for explanations.

Among SwAsian dogs, 97.4% carried a haplotype grouping within one of the three haplogroups of the universal gene pool, and 2.6% carried haplotypes grouping with sub-haplogroup d2. The proportions of individuals carrying haplogroups A, B, and C were similar to those found in earlier studies, with SwAsia having an elevated frequency of haplogroup B (33.0%) compared to other regions (Table 1). The frequency of haplogroup B was especially high (62.5%) in SwAsian sighthounds, among which almost only haplogroups A and B were present. Exclusion of this special group of breeds gave proportions of the haplogroups more in accordance with other regions of the Old World, with a frequency of haplogroup B (27.3%) slightly higher than in, for example, Europe (20.6%) and Siberia (21.0%). Exclusion of sighthounds from the SwAsian sample did not change values for the diversity measures considerably (Table 1). The frequency of SwAsian individuals having a UT and a UTd was 77.7% and 94.5%, respectively (Table 1); the concentration of haplotypes around the UTs is strikingly illustrated in the MS networks of Figs 2A and 3. This implies that 94.5% of the SwAsian dogs had a haplotype that is identical to, or differs by a single substitution from, a haplotype found across Eurasia, in both Europe and East Asia, demonstrating the homogeneity of the Eurasian gene pool. The values for UT and UTd in SwAsia are similar to those of all other populations across the Old World, except ASY (Table 1). The exceptionally low values in ASY for UT (40.5%) and UTd (52.5%) are illustrated in the MS networks of Figure 4, showing extensive representation of haplotypes on large distance from the UTs, corresponding to sub-haplogroups a2–a6. Thus, the improved sampling of SwAsian dogs did not alter the mtDNA diversity values considerably, and did not bring out evidence that the universal gene pool originated in SwAsia.

Since relatively few samples from Africa were included in the study, and considering the assertion in Boyko et al. (2009) that African village dogs have mtDNA diversity similar to that of East Asia, we compared the African dataset in Boyko et al. (2009) (Table S4) to the samples of the present study. This showed that all measures of diversity for the African village dogs were much lower than for ASY (Table S5; Fig. S1); there were, for example, 88 haplotypes among 341 samples from ASY to compare with 42 haplotypes among 309 African samples. The African sample had diversity values typical for the other parts of the Old World, especially similar to those of South Europe (Table S5), the only clear difference being a representation of seven instead of four of the 10 sub-haplogroups (Fig. S1).

### Sub-populations of SwAsia and their characteristics

The two specific SwAsian sub-samples from the Persian Plateau (*n* = 169) and Anatolia (*n* = 111) also had diversity measures that followed the general pattern found across the Old World, but with an elevated frequency of haplogroup B (Table 1). Both regions had diversity values lower than those of ASY, but similar to those of other Eurasian populations. Interestingly, there was limited sharing of haplotypes between the two regions (Fig. 4). Only eight of the 15 UTs and totally 12 haplotypes were shared, and there were no haplotypes shared exclusively between the two regions (Fig. 4; Table S1). Thus, the two gene pools were similar, but showed no signs of extensive reciprocal gene flow.

We also specifically studied samples from FC (*n* = 57), and from its adjacent areas in which much of the farm animal domestication took place, and which were part of the earliest Neolithic culture (FC-belt; *n* = 94), giving a total of 151 samples from the FC-xtd (Fig. 1). For these regions, the gene pools deviated more substantially than SwAsia in general in the representation and frequency of the haplogroups (Table 1, Fig. 4). The sample from FC had almost exclusively haplotypes belonging to haplogroups A and B, with only one individual from the universal haplogroup C, and no representation of sub-haplogroup d2. Accordingly, of the ten sub-haplogroups of the universal gene pool, only four (a1, b1, b2, and c1) were found in FC and diversity values were low. Most importantly, the frequency of haplogroup B (45.6%) was almost as high as that of haplogroup A (52.6%). FC-xtd had haplotypes belonging to haplogroup C and sub-haplogroup d2, as well as haplogroups A and B, and diversity values similar to those of the general SwAsian sample, but also here the frequency of haplogroup B (43.7%) was close to that of haplogroup A (48.3%). Thus, within the region where farming and farm animal domestication first took place, the dog mtDNA diversity was as limited as in SwAsia in general, but the gene pools of FC and FC-belt were distinguished by a frequency of haplogroup B close to 50%, which is considerably higher than in any other region.

The sighthounds in SwAsia had a similar deviation from the normal proportions of haplogroups as dogs in FC-xtd, with 33.9% haplogroup A, 62.5% haplogroup B, and almost totally lacked haplogroups C and D, in agreement with an origin from the local dog population. Interestingly, the same pattern was observed for the Canaan dog (A: 51.6%, B: 48.4%) that originates from the FC (Levine 2003). However, the sharing of haplotypes between the two groups of dogs was small: of the 14 haplotypes among sighthounds and seven among Canaan dogs, only the two UTs A11 and B1 were shared.

### Haplogroup B: more frequent, but not more diverse, in SwAsia

The similar proportions of haplogroups A, B, and C across Eurasia (Table 1) is a strong indication that these three dog haplogroups originated at a single time and place. Separate origins (starting with 100% of each haplogroup in separate centers of origin) would have demanded extreme migration rates for the universally almost identical proportions of the three haplogroups to occur among today's dog populations (Pang et al. 2009). However, the high frequency of haplogroup B in FC and FC-belt, and among SwAsian sighthounds, suggests the possibility that haplogroup B initially held a frequency of 100% within the FC-xtd region, before the introduction of haplogroups A and C from elsewhere. This poses a possibility for a separate domestication of wolf in SwAsia. However, if this was the case, it would be expected that the primary center of diversity for haplogroup B would also lie within this region.

We, therefore, compared the diversity among regions specifically for haplogroup B. ASY had the broadest coverage of the diversity for haplogroup B, with, for example, most of the haplotypes in sub-haplogroup b2 represented exclusively in ASY (Fig. 2B), and the largest number of haplogroup B haplotypes per individual carrying haplogroup B (Table 2). For SwAsia, the haplotypes were all arranged around the central haplotype B1, and only three haplotypes belonged to sub-haplogroup b2. Thus, the diversity for haplogroup B was lower for SwAsia, FC-xtd, and FC than for ASY. Accordingly, if haplogroup B had a single geographical origin, the more probable center of origin would be ASY. We considered also the possibility of separate origins of sub-haplogroups b1 and b2, although two independent origins from wolves residing in different regions but having almost identical mtDNA haplotypes seems unlikely. Strikingly, the diversity for sub-haplogroup b1 was significantly lower in ASY than in most other regions, suggesting the possibility of an origin outside ASY. However, diversity values were not highest in FC or FC-xtd, but in South Europe and Japan, and values were generally similar among regions across Eurasia. To conclude, FC and FC-xtd had the highest frequency of haplogroup B, but did not represent a center of diversity. It is therefore not strongly indicated that the high frequency of haplogroup B was caused by an independent domestication of dogs in SwAsia, rather than by genetic drift.

**Table 2:** Detailed structure and diversity information for haplogroup B in different regions. See Table 1 for more explanations

	Haplogroup B								Sub-haplogroup b1					
		
Population	n^1^	nHT	nHTuq	subHG^2^	nHTrsp24^3^	HTdiv (SD)	%UT	%UTd	n^4^	nHT	nHTrsp20^5^	HTdiv (SD)	%UT	%UTd
Total	368	29	-	b1, b2	5.62	0.469 (0.032)	82.6	97.3	340	20	3.86	0.379 (0.032)	89.4	99.7
SwAsia	114	12	6	b1, b2	5.05	0.479 (0.055)	82.5	98.2	102	9	3.43	0.356 (0.057)	92.2	100.0
Persian Plateau	46	5	1	b1, b2	3.83	0.476 (0.074)	91.3	95.6	44	4	2.91	0.428 (0.074)	95.4	100.0
Anatolia	35	6	3	b1, b2	4.72	0.360 (0.102)	82.9	100.0	32	5	3.45	0.238 (0.099)	90.6	100.0
Fertile Crescent	26	5	2	b1, b2	4.91	0.625 (0.087)	65.4	100.0	20	4	4.00	0.437 (0.130)	85.0	100.0
FC-belt	40	6	1	b1, b2	4.67	0.459 (0.090)	87.5	97.5	37	4	3.09	0.368 (0.090)	94.6	100.0
FC-xtd	66	8	3	b1, b2	5.02	0.532 (0.067)	78.8	98.5	57	6	3.57	0.389 (0.076)	91.2	100.0
SwAexclFC-xtd	48	8	3	b1, b2	4.97	0.401 (0.087)	87.5	97.9	45	5	3.27	0.317 (0.085)	93.3	100.0
SwAexclSight	79	10	5	b1, b2	5.56	0.596 (0.056)	77.2	97.5	68	8	3.94	0.470 (0.065)	89.7	100.0
SwAsianSight	35	3	1	b1, b2	2.37	0.113 (0.072)	94.3	100.0	34	2	1.58	0.059 (0.055)	97.1	100.0
Europe	95	8	3	b1	4.17	0.381 (0.060)	89.5	98.9	95	8	3.79	0.381 (0.060)	89.5	98.9
NorthContErp	30	3	0	b1	2.96	0.497 (0.080)	93.3	100.0	30	3	2.90	0.497 (0.080)	93.3	100.0
South Europe	25	6	2	b1	5.82	0.427 (0.122)	84.0	96.0	25	6	5.19	0.427 (0.122)	84.0	96.0
Africa	7	2	0	b1	–	0.286 (0.196)	100.0	100.0	7	2	–	0.286 (0.196)	100.0	100.0
Indian Subcont	5	1	0	b1	–	0.000 (0.000)	100.0	100.0	5	1	–	0.000 (0.000)	100.0	100.0
Siberia	13	3	1	b1	–	0.410 (0.154)	92.3	100.0	13	3	–	0.410 (0.154)	92.3	100.0
Japan	24	5	0	b1, b2	5	0.768 (0.048)	41.7	100.0	20	4	4.00	0.695 (0.062)	50.0	100.0
Northern China	52	5	3	b1	3.41	0.407 (0.073)	94.2	100.0	52	5	3.17	0.407 (0.073)	94.2	100.0
ASY	48	10	6	b1, b2	6.52	0.469 (0.090)	72.9	85.4	37	2	1.80	0.105 (0.066)	94.6	100.0
East Asia	133	17	12	b1, b2	6.49	0.549 (0.050)	75.2	94.7	117	9	4.080	0.421 (0.055)	85.5	100.0
West Eurasia	209	16	10	b1, b2	4.88	0.436 (0.041)	85.6	98.6	197	13	3.68	0.367 (0.042)	90.9	99.5

1 Number of samples in haplogroup B.

2 Sub-haplogroups of the haplogroup B found in the region.

3 Number of haplotypes obtained from resampling of size 24 (the size of the smallest informative sample) with 1000 replications.

4 Number of samples in sub-haplogroup b1 of the haplogroup B.

5 Number of haplotypes obtained from resampling of size 20 (the size of the smallest informative sample) with 1000 replications.

### sub-haplogroup d2: probable origin from dog-wolf crossbreeding in SwAsia

Another possible sign for a separate domestication of wolf in SwAsia, or alternatively a dog–wolf crossbreeding, is the presence of sub-haplogroup d2 only in SwAsia and the Mediterranean basin. Only 2.6% of the SwAsian dogs carried haplotypes of sub-haplogroup d2, it was not found in FC, and at 1.3% frequency in FC-xtd. If sub-haplogroup d2 would have originated through a separate domestication in SwAsia, a considerably higher frequency would be expected. Therefore, dog–wolf crossbreeding seems the probable source for this haplogroup. Three of the four d2 haplotypes were found in SwAsia, and only a single one in Iberia and North Africa (Fig. 4; Table S1), indicating SwAsia as the probable center of origin. In SwAsia, d2 was found among working mastiffs (e.g., Kangal and Torkaman) and one sighthound (Persian Greyhound), in North Africa in one sighthound (Azawakh), and in Iberia among both mastiffs (Estrela) and Sighthounds (Galgo).

## Discussion

With this study, we present the first comprehensive investigation of genetic diversity among dogs in SwAsia, the perhaps most important center of animal and plant domestication. Through extensive sampling of indigenous dogs across Turkey and Iran, and in the FC with adjacent areas, a partially blank spot in the global mtDNA phylogeography for dogs is filled in. This was of special importance because studies of archaeological data (Dayan 1994) and autosomal SNPs (vonHoldt et al. 2010) have suggested that the domestic dog may have originated in SwAsia, while studies of mtDNA have ruled out SwAsia, and instead indicated southern East Asia as the sole region of dog origins, but had limited geographical coverage of SwAsia. However, we here confirm that mtDNA data give no distinct indication that dogs originated in SwAsia through independent domestication of wolf.

Our study shows clearly that the SwAsian dog mtDNA gene pool is part of a very homogenous universal gene pool that involves all dog populations worldwide, but that the SwAsian population carries only a subset of the total phylogenetic diversity. Since only five of the total 10 principal sub-haplogroups were found in SwAsia, it is clear that the full genetic diversity of the universal gene pool cannot have originated in this region. Instead, ASY remains the only region where all 10 principal sub-haplogroups, and therefore virtually the full extent of genetic variation, have been found, strongly indicating this to be the center of origin for modern dogs.

Thus, there is no indication that the entire universal mtDNA gene pool may have originated in SwAsia. Neither is there any indication that a subset of this gene pool originated through independent domestication in SwAsia. The slightly higher frequency of haplogroup B than in other regions was not paralleled by a higher genetic diversity for clade B, or by a decrease in the frequency of haplogroup B with increasing distance from SwAsia, as would have been anticipated if haplogroup B had originated through independent domestication of wolf, or through dog–wolf crossbreeding in SwAsia. Instead, the simplest explanation for the high frequency of haplogroup B would be genetic drift at the original formation of the SwAsian dog population, by immigrating dogs carrying the three universal haplogroups.

In contrast, there are clear indications that sub-haplogroup d2 had a separate origin in SwAsia, yet through dog–wolf hybridization rather than domestication, considering the low frequency. The region of origin for d2 cannot be definitely established, because few samples carrying d2 haplotypes have been identified so far. However, since three of the four d2 haplotypes were found in SwAsia, and only a single one in the Iberian Peninsula and North Africa, and also considering the numerous human migrations along the SwAsian–Mediterranean route through history, SwAsia seems the likely region of origin for sub-haplogroup d2. Importantly, if this gene flow between dog and wolf was reciprocal (Anderson et al. 2009), this would also explain why wolves from the Middle East share more autosomal multilocus haplotypes with dogs than do wolves from Northern China, Europe, and America, as reported by vonHoldt et al. (2010).

It is important to note that in the present study, dogs from SwAsia and ASY were represented by very similar samples, representing the indigenous working dog populations of each region and containing approximately equal number of individuals, minimizing possible bias in comparisons of the two regions. It is also worth emphasizing that African dogs do not have mtDNA diversity similar to that of East Asia, as asserted in Boyko et al. (2009). Instead, diversity values for the African sample is typical for the Old World outside ASY (Table S5; Fig. S1), establishing that ASY is the only region that harbors the full extent of phylogenetic diversity of the universal dog gene pool.

The fact that other studies have indicated SwAsia as the probable region for dog origins can probably be attributed to geographical bias, since samples from ASY were largely lacking in these studies. In archaeology, SwAsia is one of the most extensively studied regions of the world, while in ASY excavations have been much less frequent. Similarly, in the recent study of genome-wide autosomal SNPs (vonHoldt et al. 2010), in which haplotypes from wolves and dogs were compared to identify the source wolf population, wolf samples from ASY were totally lacking, implying that if dogs originated in ASY it could not have been detected by this study. In addition, the SNPs used for screening were almost exclusively drawn from only two European dogs, which introduces strong ascertainment bias into the comparison of genetic diversity among geographical regions (Albrechtsen et al. 2010). Importantly, if the samples from ASY are excluded from the mtDNA dataset, there are only quite subtle difference in genetic diversity among the other regions, for example, comparing Northern China with Europe or SwAsia (Table 1); it is specifically ASY that is distinguished as the only region with practically full extent of phylogenetic diversity and, consequently, much higher diversity than all other regions. It is therefore clear that for studying dog origins, a comprehensive dataset from across the extent of the original wolf distribution is necessary, or incorrect conclusions may be drawn. In the light of the very distinct results for mtDNA, representation of samples from ASY is especially important.

Obviously, mtDNA is a single genetic marker inherited only through the female line. This leaves open the possibility that the phylogeographical pattern observed for mtDNA may reflect stochastic events, selection, or sex bias rather than dog population history. Consequently, datasets including samples from ASY need to be analyzed for additional independent markers inherited through the male line, to confirm or refute the mtDNA-based results. However, so far, mtDNA is the only dataset analyzed for a global sample of dogs or wolves, lending the picture of dog origins derived from this marker special weight.

The lack of geographical differentiation in the present dataset limits the possibility for studying migrations, but the data nevertheless give some clues. The samples from the Persian Plateau and Anatolia had limited sharing of haplotypes other than the 15 UTs. It therefore seems that haplotypes derived from the UTs in the respective areas have had limited reciprocal spread, indicating that the mountain ranges separating the two zones have formed a relatively effective barrier for the free flow of the genetic material. Furthermore, if the dog originated in East Asia, SwAsia may have mediated the pass of dogs on their spread to Europe, while an alternative route would have been via Siberia. We therefore compared the haplotype pools for SwAsia, Europe, and Siberia, to look for patterns indicating the route of migrations to Europe. Continental Europe and SwAsia shared 16 haplotypes that were not found in Siberia, while only two haplotypes not present in SwAsia were shared between Europe and Siberia (Table S6), indicating closer ties of European dogs to SwAsia than to Siberia. This may indicate that the European dog population was formed predominantly through migrations via SwAsia rather than Siberia, in agreement with suggested origins and routes of human migration, and the formation of Neolithic populations in Europe (Bramanti et al. 2009; Haak et al. 2010). Also, the sharing of sub-haplogroup d2 with North Africa and South Europe suggests a gene flow along the Mediterranean basin, probably in connection with the spread of the Neolithic (Diamond and Bellwood 2003) or more recent events such as the Muslim expansion.

It is noticeable that the FC-xtd region had especially high frequency of haplogroup B with roughly half of the individuals carrying haplogroup B, and that also SwAsian sighthounds deviated with a haplogroup B frequency of 62.5%. This gives an indication that sighthounds originated in FC, as previously speculated based on archaeological evidence (Przezdziecki et al. 2001). The sighthounds had low genetic diversity and carried only four of the 15 UTs, possibly reflecting a bottleneck in the founder population and subsequent genetic drift due to selection for certain traits. Interestingly, except two UTs, there was no sharing of haplotypes between the two main sighthound stocks of Persian Greyhound/Saluki/Tazi in the west, and Afghan Hound/Taigan/Kalagh-Tazi/Bakhmul in the east of the SwAsian region (Table S7). A similar separation is observed also compared to sighthound breeds from other parts of the Old World, which share few haplotypes with the SwAsian sighthounds. More detailed studies of sighthounds, with more comprehensive coverage of different geographical regions and morphological types, as well as more informative markers, for example, nuclear microsatellites and SNPs (Parker et al. 2004; vonHoldt et al. 2010) are required to further shed light on the apparently long history of this family of dogs.

## Data Accessibility

DNA sequences: Genbank accessions HQ261489, HQ452418–HQ452423, HQ452432, HQ452433, HQ452466–HQ452477.

## Supporting Information

**Table S1.** Total sample list, specifying haplotype, haplotype ignoring indels, sub-haplogroup, location and breed/ ecotype/function information.

**Table S2.** Definitions for the geographical study domains.

**Table S3.** Samples with new haplotypes identified among the 325 new samples sequenced in the present study.

**Table S4.** Detailed information for the African samples reported by Boyko et al. (2009).

**Table S5.** Detailed structure and diversity information for the African samples reported by Boyko et al. (2009), in comparison with other regions; see Table 1 for explanations.

**Table S6.** Detailed information on sharing of haplotypes for SwAsia, Europe, and Siberia.

**Table S7.** Detailed information on sharing of haplotypes among sighthound groups.

**Figure S1.** MS networks for the African samples reported by Boyko et al. (2009).

**Figure S2.** Topology of the MS networks and the subhaplogroups, with haplotype numbers shown.
