# Supplementary material for: Comprehensive study of mtDNA among Southwest Asian dogs contradicts independent domestication of wolf, but implies dog–wolf hybridization
Source: Ecol Evol. 2011 Nov;1(3):373–85. doi: 10.1002/ece3.35 (PMC3287314; doi:10.1002/ece3.35)
Supplement: Supplementary file 1 [file ece30001-0373-SD1.doc]

**Supporting information**

Table S1: Total sample list, listing haplotype, haplotype ignoring indels, sub-haplogroup, location and breed/ecotype information. Separate Excel sheet. the new samples, sequenced in the present study, are shaded grey.

Table S2: Definitions for the geographical study domains. Samples were not necessarily available from every sub-region included.

| Region | Definition |
| --- | --- |
| Southwest Asia | Persian Plateau, Anatolia, Scythia, Levant, Arabia |
| Persian Plateau | Iran, Afghanistan, West Pakistan, Caucasus |
| Anatolia | Asian Turkey |
| Caucasus | Azerbaijan, Armenia, Georgia |
| Arabia | Kuwait, Saudi Arabia, Yemen, Oman, UAE, Qatar, Bahrain |
| Levant | Jordan, Palestine, Lebanon, Syria |
| Scythia | Kyrgyzstan, Kazakhstan, Uzbekistan, Turkmenistan, China Xinjiang, Tajikistan |
| Fertile Crescent | Region stretching from the Persian Gulf, along western Zagros Mountains and southern Taurus Mountains, to the eastern Mediterranean coast, confined from the south by the Syrian Desert. See map in Figure 2. |
| FC-belt | Mountainous area surrounding FC along the north and east of it. See map in Figure 2. |
| FC-extended | West Iran, East Turkey, Iraq, Syria, Lebanon, Palestine, Jordan |
| Europe | North Continental Europe, South Europe, British Isles, Scandinavia |
| South Europe | Moldova, Romania, Hungary, Slovakia, Serbia, Bulgaria, Bosnia, Montenegro, Macedonia, Greece, Albania, Croatia, Slovenia, Italy, Spain, Portugal, Malta |
| North Continental Europe | Poland, Czech Republic, Austria, Switzerland, Germany, France, Netherlands, Belgium, Russia, Estonia, Latvia, Lithuania, Belarus, Ukraine |
| Africa | The whole African continent |
| Indian subcontinent | India, Bangladesh, Bhutan, Nepal, East Pakistan |
| Siberia | Non-European Russia |
| Australasia | Indonesia, Papua New Guinea, Australia, New Zealand, Malaysia, East Timor |
| Americas | The whole American continent |
| Asia South of Yangtze | Southern China, Myanmar, Laos, Thailand, Cambodia, Vietnam |
| Southern China | Yunnan, Guizhou, Guangxi, Hunan, Guangdong, Jiangxi, Fujian, Zhejiang |
| Northern China | China excluding southern China, Hainan, and Xinjiang |
| East Asia | Japan, Korea, Northern China, Asia South of Yangtze, China Hainan, Mongolia |
| West Eurasia | Southwest Asia, Europe |

Table S3: Samples with new haplotypes identified among the 325 new samples sequenced in the present study. Individuals carrying new haplotypes that are equivalent to other haplotypes when indels are ignored are shown in grey shade.

| Individual | HT | Ign Indels | subHG | Designation | Location | Area | Country | Region | Study Domain | Family | Breed/Ecotype/Function |
| --- | --- | --- | --- | --- | --- | --- | --- | --- | --- | --- | --- |
| 33IG | A 167 | A 020 | a1 | FCbelt | Igdir |  | Turkey | Anatolia | Southwest Asia |  | Kars |
| z1536 | A 167 | A 020 | a1 | FC | Dezful | Xuzestan | Iran | Persian Plateau | Southwest Asia |  | Baxtiyari |
| z1542 | A 167 | A 020 | a1 | FC | Susangerd | Xuzestan | Iran | Persian Plateau | Southwest Asia |  | Guard dog |
| z291 | A 168 | A 168 | a1 |  |  |  |  | N Cont Europe | Europe | Sighthound | Chart Polski |
| 24T | A 169 | A 169 | NA | FCbelt | Tatköy |  | Turkey | Anatolia | Southwest Asia |  |  |
| z494 | A 170 | A 170 | a1 |  |  |  | Russia | N Cont Europe | Europe | Sighthound | Stepnaya |
| con80 | A 171 | A 171 | a1 | FCbelt |  | Markazi | Iran | Persian Plateau | Southwest Asia |  | Guard dog |
| con85 | A 172 | A 172 | NA |  |  | Kerman | Iran | Persian Plateau | Southwest Asia |  | Guard dog |
| z106 | A 173 | A 173 | a1 |  |  |  | Italy | South Europe | Europe |  | Volpino Italiano |
| m345 | A 174 | A 174 | a1 |  |  |  | Spain | South Europe | Europe |  | Podenco Ibicenco |
| m618 | A 175 | A 175 | a1 |  |  |  | Italy | South Europe | Europe |  | Cirneco del Etna |
| z1011 | A 175 | A 175 | a1 |  |  |  | Italy | South Europe | Europe |  | Cirneco del Etna |
| z1013 | A 175 | A 175 | a1 |  |  |  | Italy | South Europe | Europe |  | Cirneco del Etna |
| m621 | A 176 | A 176 | a1 |  |  |  |  | South Europe | Europe |  | Dalmatian |
| z148 | A 177 | A 177 | a1 |  |  |  | Turkey | Anatolia | Southwest Asia |  | Kangal |
| m757 | A 177 | A 177 | a1 | FC |  |  | Syria | Levant | Southwest Asia |  | Kangal |
| z1537 | A 275 | A 275 | a1 | FCbelt | Ardabil | Ardabil | Iran | Persian Plateau | Southwest Asia |  | Sarabi |
| 45C | B 034 | B 034 | b1 | FCbelt | Çatalhöyük |  | Turkey | Anatolia | Southwest Asia |  |  |
| 5A | B 035 | B 035 | b1 |  | Afyon |  | Turkey | Anatolia | Southwest Asia |  |  |
| z696 | B 036 | B 036 | b1 |  |  |  | Hungary | South Europe | Europe |  | Pumi |
| con64 | B 037 | B 036 | b1 | FCbelt |  | Hormozgan | Iran | Persian Plateau | Southwest Asia |  | Sheep dog |
| m602 | B 038 | B 006 | b1 |  |  |  | Spain | South Europe | Europe |  | Podenco Ibicenco |
| m652 | B 039 | B 039 | b1 | FC |  |  | Israel | Levant | Southwest Asia |  | Canaan |
| m653 | B 039 | B 039 | b1 | FC |  |  | Israel | Levant | Southwest Asia |  | Canaan |
| 1A | B 040 | B 040 | b1 | FC | Antep |  | Turkey | Anatolia | Southwest Asia |  |  |
| 18Ka | B 041 | B 009 | b2 | FCbelt | Kars |  | Turkey | Anatolia | Southwest Asia |  | Kars |
| 40V | B 041 | B 009 | b2 | FCbelt | Van |  | Turkey | Anatolia | Southwest Asia |  | Kars |
| 59K | B 041 | B 009 | b2 |  |  |  | Turkey | Anatolia | Southwest Asia |  | Kars |
| z1527 | D 010 | D 010 | d2 |  | Zabol | Sistan | Iran | Persian Plateau | Southwest Asia | Sighthound | Persian Greyhound |

Table S4: Detailed information for the African samples reported by Boyko *et al.* (2009). Haplotype names were adopted in accordance with the nomenclature from Savolainen *et al.* (2002) and Pang *et al.* (2009), in order to adjust samples to the context of this study.

| Individual | HT | HT ignoring Indels | subHG | Original Label | GenBank Acc. No. | Location | Area | Country | Region | Type |
| --- | --- | --- | --- | --- | --- | --- | --- | --- | --- | --- |
| Boyko001 | A 001 | A 001 | a1 | vilA27 | GQ375191 | Bunanimi | Uganda mainland | Uganda | S Africa |  |
| Boyko004 | A 002 | A 002 | a1 | vilA25 | GQ375189 | Busoba | Uganda mainland | Uganda | S Africa |  |
| Boyko005 | A 002 | A 002 | a1 |  |  | Nugiso | Uganda mainland | Uganda | S Africa |  |
| Boyko006 | A 002 | A 002 | a1 |  |  | Nugiso | Uganda mainland | Uganda | S Africa |  |
| Boyko008 | A 003 | A 003 | a1 | vilA23 | GQ375187 | Butandiga | Uganda mainland | Uganda | S Africa |  |
| Boyko009 | A 003 | A 003 | a1 |  |  | Mwoma (Ddamba Island) | Uganda isles | Uganda | S Africa |  |
| Boyko010 | A 003 | A 003 | a1 |  |  | Mwoma (Ddamba Island) | Uganda isles | Uganda | S Africa |  |
| Boyko011 | A 003 | A 003 | a1 |  |  | Zingoola (Koome Island) | Uganda isles | Uganda | S Africa |  |
| Boyko012 | A 003 | A 003 | a1 |  |  | Omavela | N Namibia | Namibia | S Africa | Village dog |
| Boyko013 | A 005 | A 005 | a1 | vilA6 | GQ375169 | ACE (Animal Care in Egypt) | Luxor | Egypt | N Africa | Village dog |
| Boyko014 | A 005 | A 005 | a1 |  |  | La Galta | Luxor | Egypt | N Africa | Village dog |
| Boyko015 | A 005 | A 005 | a1 |  |  | Walchwaba | Uganda mainland | Uganda | S Africa |  |
| Boyko016 | A 005 | A 005 | a1 |  |  | Mooni | Uganda mainland | Uganda | S Africa |  |
| Boyko017 | A 005 | A 005 | a1 |  |  | Mooni | Uganda mainland | Uganda | S Africa |  |
| Boyko018 | A 005 | A 005 | a1 |  |  | Busoba | Uganda mainland | Uganda | S Africa |  |
| Boyko019 | A 005 | A 005 | a1 |  |  | Busoba | Uganda mainland | Uganda | S Africa |  |
| Boyko020 | A 005 | A 005 | a1 |  |  | Busoba | Uganda mainland | Uganda | S Africa |  |
| Boyko021 | A 005 | A 005 | a1 |  |  | Busoba | Uganda mainland | Uganda | S Africa |  |
| Boyko022 | A 005 | A 005 | a1 |  |  | Butandiga | Uganda mainland | Uganda | S Africa |  |
| Boyko023 | A 005 | A 005 | a1 |  |  | Kongoidi | Uganda mainland | Uganda | S Africa |  |
| Boyko024 | A 005 | A 005 | a1 |  |  | Kongoidi | Uganda mainland | Uganda | S Africa |  |
| Boyko025 | A 005 | A 005 | a1 |  |  | Kongoidi | Uganda mainland | Uganda | S Africa |  |
| Boyko026 | A 005 | A 005 | a1 |  |  | Kongoidi | Uganda mainland | Uganda | S Africa |  |
| Boyko027 | A 005 | A 005 | a1 |  |  | Komosingo | Uganda mainland | Uganda | S Africa |  |
| Boyko028 | A 005 | A 005 | a1 |  |  | Komosingo | Uganda mainland | Uganda | S Africa |  |
| Boyko029 | A 005 | A 005 | a1 |  |  | Komosingo | Uganda mainland | Uganda | S Africa |  |
| Boyko030 | A 005 | A 005 | a1 |  |  | Komosingo | Uganda mainland | Uganda | S Africa |  |
| Boyko031 | A 005 | A 005 | a1 |  |  | Komosingo | Uganda mainland | Uganda | S Africa |  |
| Boyko032 | A 005 | A 005 | a1 |  |  | Komosingo | Uganda mainland | Uganda | S Africa |  |
| Boyko033 | A 005 | A 005 | a1 |  |  | Komosingo | Uganda mainland | Uganda | S Africa |  |
| Boyko034 | A 005 | A 005 | a1 |  |  | Komosingo | Uganda mainland | Uganda | S Africa |  |
| Boyko035 | A 005 | A 005 | a1 |  |  | Tabaliro (Nsadzi Island) | Uganda isles | Uganda | S Africa |  |
| Boyko036 | A 005 | A 005 | a1 |  |  | Olund | N Namibia | Namibia | S Africa | Village dog |
| Boyko037 | A 011 | A 011 | a1 | vilA11a | GQ375174 | Animal Friends Shelter | Giza | Egypt | N Africa | Village dog |
| Boyko038 | A 011 | A 011 | a1 |  |  | Napoli | Uganda mainland | Uganda | S Africa |  |
| Boyko039 | A 011 | A 011 | a1 |  |  | Napoli | Uganda mainland | Uganda | S Africa |  |
| Boyko040 | A 011 | A 011 | a1 |  |  | Walchwaba | Uganda mainland | Uganda | S Africa |  |
| Boyko041 | A 011 | A 011 | a1 |  |  | Walchwaba | Uganda mainland | Uganda | S Africa |  |
| Boyko042 | A 011 | A 011 | a1 |  |  | Namabasa | Uganda mainland | Uganda | S Africa |  |
| Boyko043 | A 011 | A 011 | a1 |  |  | Namabasa | Uganda mainland | Uganda | S Africa |  |
| Boyko044 | A 011 | A 011 | a1 |  |  | Namabasa | Uganda mainland | Uganda | S Africa |  |
| Boyko045 | A 011 | A 011 | a1 |  |  | Namabasa | Uganda mainland | Uganda | S Africa |  |
| Boyko046 | A 011 | A 011 | a1 |  |  | Mooni | Uganda mainland | Uganda | S Africa |  |
| Boyko047 | A 011 | A 011 | a1 |  |  | Mooni | Uganda mainland | Uganda | S Africa |  |
| Boyko048 | A 011 | A 011 | a1 |  |  | Mooni | Uganda mainland | Uganda | S Africa |  |
| Boyko049 | A 011 | A 011 | a1 |  |  | Bunanimi | Uganda mainland | Uganda | S Africa |  |
| Boyko050 | A 011 | A 011 | a1 |  |  | Bunanimi | Uganda mainland | Uganda | S Africa |  |
| Boyko051 | A 011 | A 011 | a1 |  |  | Bunanimi | Uganda mainland | Uganda | S Africa |  |
| Boyko052 | A 011 | A 011 | a1 |  |  | Komosingo | Uganda mainland | Uganda | S Africa |  |
| Boyko053 | A 011 | A 011 | a1 |  |  | Komosingo | Uganda mainland | Uganda | S Africa |  |
| Boyko054 | A 011 | A 011 | a1 |  |  | Zingoola (Koome Island) | Uganda isles | Uganda | S Africa |  |
| Boyko055 | A 011 | A 011 | a1 |  |  | Grootfontaine | C Namibia | Namibia | S Africa | Village dog |
| Boyko056 | A 011 | A 011 | a1 |  |  | Grootfontaine | C Namibia | Namibia | S Africa | Village dog |
| Boyko057 | A 011 | A 011 | a1 |  |  | Grootfontaine | C Namibia | Namibia | S Africa | Village dog |
| Boyko058 | A 011 | A 011 | a1 |  |  | Grootfontaine | C Namibia | Namibia | S Africa | Village dog |
| Boyko059 | A 011 | A 011 | a1 |  |  | Oshikango | N Namibia | Namibia | S Africa | Village dog |
| Boyko060 | A 011 | A 011 | a1 |  |  | Ongha | N Namibia | Namibia | S Africa | Village dog |
| Boyko061 | A 011 | A 011 | a1 |  |  | Indiangungu | N Namibia | Namibia | S Africa | Village dog |
| Boyko062 | A 011 | A 011 | a1 |  |  | Ondandwa | N Namibia | Namibia | S Africa | Village dog |
| Boyko063 | A 011 | A 011 | a1 |  |  | Omaarara | N Namibia | Namibia | S Africa | Village dog |
| Boyko064 | A 011 | A 011 | a1 |  |  | Okanbjengedhi | N Namibia | Namibia | S Africa | Village dog |
| Boyko065 | A 011 | A 011 | a1 |  |  | Okanbjengedhi | N Namibia | Namibia | S Africa | Village dog |
| Boyko066 | A 011 | A 011 | a1 |  |  | Okanbjengedhi | N Namibia | Namibia | S Africa | Village dog |
| Boyko067 | A 011 | A 011 | a1 |  |  | Oshikuku | N Namibia | Namibia | S Africa | Village dog |
| Boyko068 | A 011 | A 011 | a1 |  |  | Omavela | N Namibia | Namibia | S Africa | Village dog |
| Boyko069 | A 011 | A 011 | a1 |  |  | Endola | N Namibia | Namibia | S Africa | Village dog |
| Boyko070 | A 011 | A 011 | a1 |  |  | Onyvulae | N Namibia | Namibia | S Africa | Village dog |
| Boyko071 | A 011 | A 011 | a1 |  |  | Onyvulae | N Namibia | Namibia | S Africa | Village dog |
| Boyko072 | A 011 | A 011 | a1 |  |  | Onyvulae | N Namibia | Namibia | S Africa | Village dog |
| Boyko076 | A 016 | A 016 | a1 | vilA16 | GQ375180 | Bunanimi | Uganda mainland | Uganda | S Africa |  |
| Boyko077 | A 016 | A 016 | a1 |  |  | Nugiso | Uganda mainland | Uganda | S Africa |  |
| Boyko078 | A 016 | A 016 | a1 |  |  | Butandiga | Uganda mainland | Uganda | S Africa |  |
| Boyko079 | A 016 | A 016 | a1 |  |  | Buchenda | Uganda mainland | Uganda | S Africa |  |
| Boyko080 | A 016 | A 016 | a1 |  |  | Grootfontaine | C Namibia | Namibia | S Africa | Village dog |
| Boyko081 | A 016 | A 016 | a1 |  |  | Grootfontaine | C Namibia | Namibia | S Africa | Village dog |
| Boyko082 | A 016 | A 016 | a1 |  |  | Grootfontaine | C Namibia | Namibia | S Africa | Village dog |
| Boyko083 | A 016 | A 016 | a1 |  |  | Grootfontaine | C Namibia | Namibia | S Africa | Village dog |
| Boyko084 | A 016 | A 016 | a1 |  |  | Ongha | N Namibia | Namibia | S Africa | Village dog |
| Boyko085 | A 016 | A 016 | a1 |  |  | Ondandwa | N Namibia | Namibia | S Africa | Village dog |
| Boyko086 | A 016 | A 016 | a1 |  |  | Cham-Cham | N Namibia | Namibia | S Africa | Village dog |
| Boyko088 | A 017 | A 017 | a1 | vilA13 | GQ375177 | Napoli | Uganda mainland | Uganda | S Africa |  |
| Boyko089 | A 017 | A 017 | a1 |  |  | Walchwaba | Uganda mainland | Uganda | S Africa |  |
| Boyko090 | A 017 | A 017 | a1 |  |  | Walchwaba | Uganda mainland | Uganda | S Africa |  |
| Boyko091 | A 017 | A 017 | a1 |  |  | Walchwaba | Uganda mainland | Uganda | S Africa |  |
| Boyko092 | A 017 | A 017 | a1 |  |  | Walchwaba | Uganda mainland | Uganda | S Africa |  |
| Boyko093 | A 017 | A 017 | a1 |  |  | Walchwaba | Uganda mainland | Uganda | S Africa |  |
| Boyko094 | A 017 | A 017 | a1 |  |  | Mooni | Uganda mainland | Uganda | S Africa |  |
| Boyko095 | A 017 | A 017 | a1 |  |  | Mooni | Uganda mainland | Uganda | S Africa |  |
| Boyko096 | A 017 | A 017 | a1 |  |  | Mooni | Uganda mainland | Uganda | S Africa |  |
| Boyko097 | A 017 | A 017 | a1 |  |  | Mooni | Uganda mainland | Uganda | S Africa |  |
| Boyko098 | A 017 | A 017 | a1 |  |  | Busoba | Uganda mainland | Uganda | S Africa |  |
| Boyko099 | A 017 | A 017 | a1 |  |  | Butandiga | Uganda mainland | Uganda | S Africa |  |
| Boyko100 | A 017 | A 017 | a1 |  |  | Otavi | C Namibia | Namibia | S Africa | Village dog |
| Boyko101 | A 017 | A 017 | a1 |  |  | Otavi | C Namibia | Namibia | S Africa | Village dog |
| Boyko102 | A 017 | A 017 | a1 |  |  | Otavi | C Namibia | Namibia | S Africa | Village dog |
| Boyko103 | A 017 | A 017 | a1 |  |  | Tsumeb | C Namibia | Namibia | S Africa | Village dog |
| Boyko104 | A 017 | A 017 | a1 |  |  | Tsumeb | C Namibia | Namibia | S Africa | Village dog |
| Boyko105 | A 017 | A 017 | a1 |  |  | Grootfontaine | C Namibia | Namibia | S Africa | Village dog |
| Boyko106 | A 017 | A 017 | a1 |  |  | Oshivelo | N Namibia | Namibia | S Africa | Village dog |
| Boyko107 | A 017 | A 017 | a1 |  |  | Oshivelo | N Namibia | Namibia | S Africa | Village dog |
| Boyko108 | A 017 | A 017 | a1 |  |  | Oshikango | N Namibia | Namibia | S Africa | Village dog |
| Boyko109 | A 017 | A 017 | a1 |  |  | Onhuno | N Namibia | Namibia | S Africa | Village dog |
| Boyko110 | A 017 | A 017 | a1 |  |  | Onhuno | N Namibia | Namibia | S Africa | Village dog |
| Boyko111 | A 017 | A 017 | a1 |  |  | Ondandwa | N Namibia | Namibia | S Africa | Village dog |
| Boyko112 | A 017 | A 017 | a1 |  |  | Omaarara | N Namibia | Namibia | S Africa | Village dog |
| Boyko113 | A 017 | A 017 | a1 |  |  | Okanbjengedhi | N Namibia | Namibia | S Africa | Village dog |
| Boyko114 | A 017 | A 017 | a1 |  |  | Okanbjengedhi | N Namibia | Namibia | S Africa | Village dog |
| Boyko115 | A 017 | A 017 | a1 |  |  | Omavela | N Namibia | Namibia | S Africa | Village dog |
| Boyko116 | A 017 | A 017 | a1 |  |  | Endola | N Namibia | Namibia | S Africa | Village dog |
| Boyko117 | A 017 | A 017 | a1 |  |  | Endola | N Namibia | Namibia | S Africa | Village dog |
| Boyko118 | A 017 | A 017 | a1 |  |  | Endola | N Namibia | Namibia | S Africa | Village dog |
| Boyko119 | A 017 | A 017 | a1 |  |  | Onyvulae | N Namibia | Namibia | S Africa | Village dog |
| Boyko120 | A 017 | A 017 | a1 |  |  | Onyvulae | N Namibia | Namibia | S Africa | Village dog |
| Boyko121 | A 017 | A 017 | a1 |  |  | Cham-Cham | N Namibia | Namibia | S Africa | Village dog |
| Boyko122 | A 017 | A 017 | a1 |  |  | Cham-Cham | N Namibia | Namibia | S Africa | Village dog |
| Boyko125 | A 018 | A 018 | a1 | vilA2 | GQ375165 | Animal Friends Shelter | Giza | Egypt | N Africa | Village dog |
| Boyko126 | A 018 | A 018 | a1 |  |  | Animal Friends Shelter | Giza | Egypt | N Africa | Village dog |
| Boyko127 | A 018 | A 018 | a1 |  |  | Animal Friends Shelter | Giza | Egypt | N Africa | Village dog |
| Boyko128 | A 018 | A 018 | a1 |  |  | ACE (Animal Care in Egypt) | Luxor | Egypt | N Africa | Village dog |
| Boyko129 | A 018 | A 018 | a1 |  |  | Napoli | Uganda mainland | Uganda | S Africa |  |
| Boyko130 | A 018 | A 018 | a1 |  |  | Busoba | Uganda mainland | Uganda | S Africa |  |
| Boyko131 | A 018 | A 018 | a1 |  |  | Bunanimi | Uganda mainland | Uganda | S Africa |  |
| Boyko132 | A 018 | A 018 | a1 |  |  | Nugiso | Uganda mainland | Uganda | S Africa |  |
| Boyko133 | A 018 | A 018 | a1 |  |  | Butandiga | Uganda mainland | Uganda | S Africa |  |
| Boyko134 | A 018 | A 018 | a1 |  |  | Kongoidi | Uganda mainland | Uganda | S Africa |  |
| Boyko135 | A 018 | A 018 | a1 |  |  | Komosingo | Uganda mainland | Uganda | S Africa |  |
| Boyko136 | A 018 | A 018 | a1 |  |  | Zingoola (Koome Island) | Uganda isles | Uganda | S Africa |  |
| Boyko137 | A 018 | A 018 | a1 |  |  | Onhuno | N Namibia | Namibia | S Africa | Village dog |
| Boyko138 | A 018 | A 018 | a1 |  |  | Onyati | N Namibia | Namibia | S Africa | Village dog |
| Boyko139 | A 018 | A 018 | a1 |  |  | Onyati | N Namibia | Namibia | S Africa | Village dog |
| Boyko140 | A 018 | A 018 | a1 |  |  | Onyati | N Namibia | Namibia | S Africa | Village dog |
| Boyko141 | A 018 | A 018 | a1 |  |  | Otavi | C Namibia | Namibia | S Africa | Village dog |
| Boyko144 | A 019 | A 019 | a1 | vilA5 | GQ375168 | Animal Friends Shelter | Giza | Egypt | N Africa | Village dog |
| Boyko145 | A 019 | A 019 | a1 |  |  | Animal Friends Shelter | Giza | Egypt | N Africa | Village dog |
| Boyko146 | A 019 | A 019 | a1 |  |  | Navigyo | Uganda mainland | Uganda | S Africa |  |
| Boyko147 | A 019 | A 019 | a1 |  |  | Navigyo | Uganda mainland | Uganda | S Africa |  |
| Boyko148 | A 019 | A 019 | a1 |  |  | Navigyo | Uganda mainland | Uganda | S Africa |  |
| Boyko149 | A 019 | A 019 | a1 |  |  | Tabaliro (Nsadzi Island) | Uganda isles | Uganda | S Africa |  |
| Boyko150 | A 019 | A 019 | a1 |  |  | Tabaliro (Nsadzi Island) | Uganda isles | Uganda | S Africa |  |
| Boyko151 | A 019 | A 019 | a1 |  |  | Tabaliro (Nsadzi Island) | Uganda isles | Uganda | S Africa |  |
| Boyko152 | A 019 | A 019 | a1 |  |  | Olund | N Namibia | Namibia | S Africa | Village dog |
| Boyko153 | A 019 | A 019 | a1 |  |  | Omaarara | N Namibia | Namibia | S Africa | Village dog |
| Boyko154 | A 019 | A 019 | a1 |  |  | Omaarara | N Namibia | Namibia | S Africa | Village dog |
| Boyko155 | A 019 | A 019 | a1 |  |  | Omaarara | N Namibia | Namibia | S Africa | Village dog |
| Boyko156 | A 019 | A 019 | a1 |  |  | Endola | N Namibia | Namibia | S Africa | Village dog |
| Boyko157 | A 020 | A 020 | a1 | vilA15 | GQ375179 | Namabasa | Uganda mainland | Uganda | S Africa |  |
| Boyko158 | A 020 | A 020 | a1 |  |  | Komosingo | Uganda mainland | Uganda | S Africa |  |
| Boyko159 | A 020 | A 020 | a1 |  |  | Oshikango | N Namibia | Namibia | S Africa | Village dog |
| Boyko160 | A 020 | A 020 | a1 |  |  | Oshikango | N Namibia | Namibia | S Africa | Village dog |
| Boyko161 | A 020 | A 020 | a1 |  |  | Oshikango | N Namibia | Namibia | S Africa | Village dog |
| Boyko162 | A 020 | A 020 | a1 |  |  | Ondandwa | N Namibia | Namibia | S Africa | Village dog |
| Boyko163 | A 020 | A 020 | a1 |  |  | Okanbjengedhi | N Namibia | Namibia | S Africa | Village dog |
| Boyko164 | A 022 | A 022 | a1 | vilA1 | GQ375164 | Animal Friends Shelter | Giza | Egypt | N Africa | Village dog |
| Boyko165 | A 022 | A 022 | a1 |  |  | Animal Friends Shelter | Giza | Egypt | N Africa | Village dog |
| Boyko166 | A 022 | A 022 | a1 |  |  | Gouahera | Luxor | Egypt | N Africa | Village dog |
| Boyko167 | A 022 | A 022 | a1 |  |  | Napoli | Uganda mainland | Uganda | S Africa |  |
| Boyko168 | A 022 | A 022 | a1 |  |  | Napoli | Uganda mainland | Uganda | S Africa |  |
| Boyko169 | A 022 | A 022 | a1 |  |  | Napoli | Uganda mainland | Uganda | S Africa |  |
| Boyko170 | A 022 | A 022 | a1 |  |  | Nugiso | Uganda mainland | Uganda | S Africa |  |
| Boyko171 | A 027 | A 027 | a1 | vilA7 | GQ375170 | Gouahera | Luxor | Egypt | N Africa | Village dog |
| Boyko172 | A 027 | A 027 | a1 |  |  | Gouahera | Luxor | Egypt | N Africa | Village dog |
| Boyko173 | A 027 | A 027 | a1 |  |  | Jazeera | Luxor | Egypt | N Africa | Village dog |
| Boyko174 | A 027 | A 027 | a1 |  |  | Butandiga | Uganda mainland | Uganda | S Africa |  |
| Boyko175 | A 027 | A 027 | a1 |  |  | Grootfontaine | C Namibia | Namibia | S Africa | Village dog |
| Boyko176 | A 027 | A 027 | a1 |  |  | Ongha | N Namibia | Namibia | S Africa | Village dog |
| Boyko177 | A 027 | A 027 | a1 |  |  | Indiangungu | N Namibia | Namibia | S Africa | Village dog |
| Boyko178 | A 027 | A 027 | a1 |  |  | Onyvulae | N Namibia | Namibia | S Africa | Village dog |
| Boyko180 | A 032 | A 032 | a1 | vilA19 | GQ375183 | Indiangungu | N Namibia | Namibia | S Africa | Village dog |
| Boyko181 | A 032 | A 032 | a1 |  |  | Olund | N Namibia | Namibia | S Africa | Village dog |
| Boyko182 | A 032 | A 032 | a1 |  |  | Olund | N Namibia | Namibia | S Africa | Village dog |
| Boyko183 | A 032 | A 032 | a1 |  |  | Omaarara | N Namibia | Namibia | S Africa | Village dog |
| Boyko184 | A 032 | A 032 | a1 |  |  | Omaarara | N Namibia | Namibia | S Africa | Village dog |
| Boyko185 | A 033 | A 033 | a1 | vilA9 | GQ375172 | Bairat | Luxor | Egypt | N Africa | Village dog |
| Boyko186 | A 038 | A 038 | a2 | vilA21 | GQ375185 | Okanbjengedhi | N Namibia | Namibia | S Africa | Village dog |
| Boyko187 | A 049 | A 049 | a1 | vilA30 | GQ375194 | Mwoma (Ddamba Island) | Uganda isles | Uganda | S Africa |  |
| Boyko188 | A 049 | A 049 | a1 |  |  | Mwoma (Ddamba Island) | Uganda isles | Uganda | S Africa |  |
| Boyko189 | A 049 | A 049 | a1 |  |  | Mwoma (Ddamba Island) | Uganda isles | Uganda | S Africa |  |
| Boyko190 | A 049 | A 049 | a1 |  |  | Mwoma (Ddamba Island) | Uganda isles | Uganda | S Africa |  |
| Boyko191 | A 049 | A 049 | a1 |  |  | Busiro (Koome Island) | Uganda isles | Uganda | S Africa |  |
| Boyko192 | A 065 | A 065 | a4 | vilA20 | GQ375184 | Ondandwa | N Namibia | Namibia | S Africa | Village dog |
| Boyko193 | A 065 | A 065 | a4 |  |  | Okanbjengedhi | N Namibia | Namibia | S Africa | Village dog |
| Boyko194 | A 065 | A 065 | a4 |  |  | Oshikuku | N Namibia | Namibia | S Africa | Village dog |
| Boyko195 | A 065 | A 065 | a4 |  |  | Endola | N Namibia | Namibia | S Africa | Village dog |
| Boyko196 | A 071 | A 071 | a1 | vilA14 | GQ375178 | Nugiso | Uganda mainland | Uganda | S Africa |  |
| Boyko197 | A 071 | A 071 | a1 |  |  | Tsumeb | C Namibia | Namibia | S Africa | Village dog |
| Boyko198 | A 071 | A 071 | a1 |  |  | Tsumeb | C Namibia | Namibia | S Africa | Village dog |
| Boyko199 | A 071 | A 071 | a1 |  |  | Tsumeb | C Namibia | Namibia | S Africa | Village dog |
| Boyko200 | A 071 | A 071 | a1 |  |  | Olund | N Namibia | Namibia | S Africa | Village dog |
| Boyko201 | A 071 | A 071 | a1 |  |  | Olund | N Namibia | Namibia | S Africa | Village dog |
| Boyko202 | A 080 | A 080 | a1 | vilA17 | GQ375181 | Onhuno | N Namibia | Namibia | S Africa | Village dog |
| Boyko203 | A 080 | A 080 | a1 |  |  | Endola | N Namibia | Namibia | S Africa | Village dog |
| Boyko204 | A 153 | A 153 | NA | vilA34 | GQ375198 | Onyvulae | N Namibia | Namibia | S Africa | Village dog |
| Boyko205 | A 169 | A 169 | NA | vilA3 | GQ375166 | ACE (Animal Care in Egypt) | Luxor | Egypt | N Africa | Village dog |
| Boyko206 | A 169 | A 169 | NA |  |  | ACE (Animal Care in Egypt) | Luxor | Egypt | N Africa | Village dog |
| Boyko207 | A 169 | A 169 | NA |  |  | ACE (Animal Care in Egypt) | Luxor | Egypt | N Africa | Village dog |
| Boyko208 | A 169 | A 169 | NA |  |  | Gouahera | Luxor | Egypt | N Africa | Village dog |
| Boyko209 | A 169 | A 169 | NA |  |  | Jazeera | Luxor | Egypt | N Africa | Village dog |
| Boyko210 | A 252 | A 252 | a1 | vilA18 | GQ375182 | Ongha | N Namibia | Namibia | S Africa | Village dog |
| Boyko211 | A 252 | A 252 | a1 |  |  | Indiangungu | N Namibia | Namibia | S Africa | Village dog |
| Boyko212 | A 253 | A 253 | a1 | vilA24 | GQ375188 | Komosingo | Uganda mainland | Uganda | S Africa |  |
| Boyko213 | A 253 | A 253 | a1 |  |  | Onyvulae | N Namibia | Namibia | S Africa | Village dog |
| Boyko297 | A 263 | A 263 | a1 | vilA4 | GQ375167 | ACE (Animal Care in Egypt) | Luxor | Egypt | N Africa | Village dog |
| Boyko298 | A 264 | A 264 | NA | vilA8 | GQ375171 | Gouahera | Luxor | Egypt | N Africa | Village dog |
| Boyko299 | A 264 | A 264 | NA |  |  | Gouahera | Luxor | Egypt | N Africa | Village dog |
| Boyko300 | A 265 | A 265 | a1 | vilA10 | GQ375173 | Bairat | Luxor | Egypt | N Africa | Village dog |
| Boyko301 | A 266 | A 011 | a1 | vilA11b | GQ375175 | Napoli | Uganda mainland | Uganda | S Africa |  |
| Boyko302 | A 266 | A 011 | a1 |  |  | Napoli | Uganda mainland | Uganda | S Africa |  |
| Boyko303 | A 266 | A 011 | a1 |  |  | Walchwaba | Uganda mainland | Uganda | S Africa |  |
| Boyko304 | A 266 | A 011 | a1 |  |  | Walchwaba | Uganda mainland | Uganda | S Africa |  |
| Boyko305 | A 267 | A 267 | a1 | vilA12 | GQ375176 | Animal Friends Shelter | Giza | Egypt | N Africa | Village dog |
| Boyko306 | A 268 | A 268 | a1 | vilA22 | GQ375186 | Oshikuku | N Namibia | Namibia | S Africa | Village dog |
| Boyko307 | A 268 | A 268 | a1 |  |  | Oshikuku | N Namibia | Namibia | S Africa | Village dog |
| Boyko308 | A 268 | A 268 | a1 |  |  | Onyvulae | N Namibia | Namibia | S Africa | Village dog |
| Boyko309 | A 269 | A 269 | NA | vilA29 | GQ375193 | Komosingo | Uganda mainland | Uganda | S Africa |  |
| Boyko310 | A 270 | A 270 | a1 | vilA31 | GQ375195 | Kisigala (Koome Island) | Uganda isles | Uganda | S Africa |  |
| Boyko311 | A 270 | A 270 | a1 |  |  | Kisigala (Koome Island) | Uganda isles | Uganda | S Africa |  |
| Boyko312 | A 270 | A 270 | a1 |  |  | Kisigala (Koome Island) | Uganda isles | Uganda | S Africa |  |
| Boyko313 | A 270 | A 270 | a1 |  |  | Kisigala (Koome Island) | Uganda isles | Uganda | S Africa |  |
| Boyko314 | A 270 | A 270 | a1 |  |  | Zingoola (Koome Island) | Uganda isles | Uganda | S Africa |  |
| Boyko315 | A 270 | A 270 | a1 |  |  | Zingoola (Koome Island) | Uganda isles | Uganda | S Africa |  |
| Boyko316 | A 270 | A 270 | a1 |  |  | Zingoola (Koome Island) | Uganda isles | Uganda | S Africa |  |
| Boyko317 | A 271 | A 271 | a1 | vilA32 | GQ375196 | Mooni | Uganda mainland | Uganda | S Africa |  |
| Boyko318 | A 271 | A 271 | a1 |  |  | Mooni | Uganda mainland | Uganda | S Africa |  |
| Boyko319 | A 271 | A 271 | a1 |  |  | Mooni | Uganda mainland | Uganda | S Africa |  |
| Boyko320 | A 271 | A 271 | a1 |  |  | Mooni | Uganda mainland | Uganda | S Africa |  |
| Boyko321 | A 271 | A 271 | a1 |  |  | Mooni | Uganda mainland | Uganda | S Africa |  |
| Boyko322 | A 271 | A 271 | a1 |  |  | Butandiga | Uganda mainland | Uganda | S Africa |  |
| Boyko323 | A 272 | A 272 | a1 | vilA33 | GQ375197 | Busoba | Uganda mainland | Uganda | S Africa |  |
| Boyko324 | A 272 | A 272 | a1 |  |  | Busoba | Uganda mainland | Uganda | S Africa |  |
| Boyko215 | B 001 | B 001 | b1 | vilB1b/vilB1c | GQ375200/GQ375201 | Ezba | Luxor | Egypt | N Africa | Village dog |
| Boyko216 | B 001 | B 001 | b1 |  |  | Comb | Luxor | Egypt | N Africa | Village dog |
| Boyko217 | B 001 | B 001 | b1 |  |  | Napoli | Uganda mainland | Uganda | S Africa |  |
| Boyko218 | B 001 | B 001 | b1 |  |  | Namabasa | Uganda mainland | Uganda | S Africa |  |
| Boyko219 | B 001 | B 001 | b1 |  |  | Namabasa | Uganda mainland | Uganda | S Africa |  |
| Boyko220 | B 001 | B 001 | b1 |  |  | Namabasa | Uganda mainland | Uganda | S Africa |  |
| Boyko221 | B 001 | B 001 | b1 |  |  | Mooni | Uganda mainland | Uganda | S Africa |  |
| Boyko222 | B 001 | B 001 | b1 |  |  | Mooni | Uganda mainland | Uganda | S Africa |  |
| Boyko223 | B 001 | B 001 | b1 |  |  | Mooni | Uganda mainland | Uganda | S Africa |  |
| Boyko224 | B 001 | B 001 | b1 |  |  | Mooni | Uganda mainland | Uganda | S Africa |  |
| Boyko225 | B 001 | B 001 | b1 |  |  | Mooni | Uganda mainland | Uganda | S Africa |  |
| Boyko226 | B 001 | B 001 | b1 |  |  | Nugiso | Uganda mainland | Uganda | S Africa |  |
| Boyko227 | B 001 | B 001 | b1 |  |  | Butandiga | Uganda mainland | Uganda | S Africa |  |
| Boyko228 | B 001 | B 001 | b1 |  |  | Butandiga | Uganda mainland | Uganda | S Africa |  |
| Boyko229 | B 001 | B 001 | b1 |  |  | Butandiga | Uganda mainland | Uganda | S Africa |  |
| Boyko230 | B 001 | B 001 | b1 |  |  | Butandiga | Uganda mainland | Uganda | S Africa |  |
| Boyko231 | B 001 | B 001 | b1 |  |  | Butandiga | Uganda mainland | Uganda | S Africa |  |
| Boyko232 | B 001 | B 001 | b1 |  |  | Butandiga | Uganda mainland | Uganda | S Africa |  |
| Boyko233 | B 001 | B 001 | b1 |  |  | Butandiga | Uganda mainland | Uganda | S Africa |  |
| Boyko234 | B 001 | B 001 | b1 |  |  | Komosingo | Uganda mainland | Uganda | S Africa |  |
| Boyko235 | B 001 | B 001 | b1 |  |  | Komosingo | Uganda mainland | Uganda | S Africa |  |
| Boyko236 | B 001 | B 001 | b1 |  |  | Komosingo | Uganda mainland | Uganda | S Africa |  |
| Boyko237 | B 001 | B 001 | b1 |  |  | Komosingo | Uganda mainland | Uganda | S Africa |  |
| Boyko238 | B 001 | B 001 | b1 |  |  | Komosingo | Uganda mainland | Uganda | S Africa |  |
| Boyko239 | B 001 | B 001 | b1 |  |  | Kongoidi | Uganda mainland | Uganda | S Africa |  |
| Boyko240 | B 001 | B 001 | b1 |  |  | Kongoidi | Uganda mainland | Uganda | S Africa |  |
| Boyko241 | B 001 | B 001 | b1 |  |  | Mwoma (Ddamba Island) | Uganda isles | Uganda | S Africa |  |
| Boyko242 | B 001 | B 001 | b1 |  |  | Busiro (Koome Island) | Uganda isles | Uganda | S Africa |  |
| Boyko243 | B 001 | B 001 | b1 |  |  | Kisigala (Koome Island) | Uganda isles | Uganda | S Africa |  |
| Boyko244 | B 001 | B 001 | b1 |  |  | Kisigala (Koome Island) | Uganda isles | Uganda | S Africa |  |
| Boyko245 | B 001 | B 001 | b1 |  |  | Zingoola (Koome Island) | Uganda isles | Uganda | S Africa |  |
| Boyko246 | B 001 | B 001 | b1 |  |  | Zingoola (Koome Island) | Uganda isles | Uganda | S Africa |  |
| Boyko247 | B 001 | B 001 | b1 |  |  | Zingoola (Koome Island) | Uganda isles | Uganda | S Africa |  |
| Boyko248 | B 001 | B 001 | b1 |  |  | Zingoola (Koome Island) | Uganda isles | Uganda | S Africa |  |
| Boyko249 | B 001 | B 001 | b1 |  |  | Zingoola (Koome Island) | Uganda isles | Uganda | S Africa |  |
| Boyko250 | B 001 | B 001 | b1 |  |  | Tsumeb | C Namibia | Namibia | S Africa | Village dog |
| Boyko251 | B 001 | B 001 | b1 |  |  | Tsumeb | C Namibia | Namibia | S Africa | Village dog |
| Boyko252 | B 001 | B 001 | b1 |  |  | Oshivelo | N Namibia | Namibia | S Africa | Village dog |
| Boyko253 | B 001 | B 001 | b1 |  |  | Oshikango | N Namibia | Namibia | S Africa | Village dog |
| Boyko254 | B 001 | B 001 | b1 |  |  | Onhuno | N Namibia | Namibia | S Africa | Village dog |
| Boyko255 | B 001 | B 001 | b1 |  |  | Onhuno | N Namibia | Namibia | S Africa | Village dog |
| Boyko256 | B 001 | B 001 | b1 |  |  | Ongha | N Namibia | Namibia | S Africa | Village dog |
| Boyko257 | B 001 | B 001 | b1 |  |  | Okanbjengedhi | N Namibia | Namibia | S Africa | Village dog |
| Boyko258 | B 001 | B 001 | b1 |  |  | Okanbjengedhi | N Namibia | Namibia | S Africa | Village dog |
| Boyko259 | B 001 | B 001 | b1 |  |  | Oshikuku | N Namibia | Namibia | S Africa | Village dog |
| Boyko260 | B 001 | B 001 | b1 |  |  | Oshikuku | N Namibia | Namibia | S Africa | Village dog |
| Boyko261 | B 001 | B 001 | b1 |  |  | Omavela | N Namibia | Namibia | S Africa | Village dog |
| Boyko266 | B 002 | B 001 | b1 | vilB1a | GQ375199 | Bagdad | Kharga | Egypt | N Africa | Village dog |
| Boyko267 | B 002 | B 001 | b1 |  |  | Bagdad | Kharga | Egypt | N Africa | Village dog |
| Boyko268 | B 002 | B 001 | b1 |  |  | Bagdad | Kharga | Egypt | N Africa | Village dog |
| Boyko269 | B 002 | B 001 | b1 |  |  | Jeddah | Kharga | Egypt | N Africa | Village dog |
| Boyko270 | B 002 | B 001 | b1 |  |  | Tsumeb | C Namibia | Namibia | S Africa | Village dog |
| Boyko271 | B 002 | B 001 | b1 |  |  | Ongha | N Namibia | Namibia | S Africa | Village dog |
| Boyko272 | B 010 | B 006 | b1 | vilB3b | GQ375204 | Ondandwa | N Namibia | Namibia | S Africa | Village dog |
| Boyko273 | B 038 | B 006 | b1 | vilB3a | GQ375203 | Indiangungu | N Namibia | Namibia | S Africa | Village dog |
| Boyko274 | B 038 | B 006 | b1 |  |  | Omavela | N Namibia | Namibia | S Africa | Village dog |
| Boyko275 | B 041 | B 009 | b2 | vilB4 | GQ375205 | Busoba | Uganda mainland | Uganda | S Africa |  |
| Boyko276 | B 041 | B 009 | b2 |  |  | Butandiga | Uganda mainland | Uganda | S Africa |  |
| Boyko325 | B 054 | B 054 | b1 | vilB2 | GQ375202 | Bagdad | Kharga | Egypt | N Africa | Village dog |
| Boyko277 | C 001 | C 001 | c1 | vilC3b | GQ375210 | Namabasa | Uganda mainland | Uganda | S Africa |  |
| Boyko278 | C 001 | C 001 | c1 |  |  | Mooni | Uganda mainland | Uganda | S Africa |  |
| Boyko279 | C 002 | C 001 | c1 | vilC3a | GQ375209 | Animal Friends Shelter | Giza | Egypt | N Africa | Village dog |
| Boyko280 | C 002 | C 001 | c1 |  |  | Otavi | C Namibia | Namibia | S Africa | Village dog |
| Boyko281 | C 002 | C 001 | c1 |  |  | Otavi | C Namibia | Namibia | S Africa | Village dog |
| Boyko282 | C 002 | C 001 | c1 |  |  | Grootfontaine | C Namibia | Namibia | S Africa | Village dog |
| Boyko283 | C 003 | C 003 | c2 | vilC2 | GQ375208 | Animal Friends Shelter | Giza | Egypt | N Africa | Village dog |
| Boyko284 | C 003 | C 003 | c2 |  |  | ACE (Animal Care in Egypt) | Luxor | Egypt | N Africa | Village dog |
| Boyko285 | C 003 | C 003 | c2 |  |  | Navigyo | Uganda mainland | Uganda | S Africa |  |
| Boyko286 | C 003 | C 003 | c2 |  |  | Tsumeb | C Namibia | Namibia | S Africa | Village dog |
| Boyko287 | C 003 | C 003 | c2 |  |  | Tsumeb | C Namibia | Namibia | S Africa | Village dog |
| Boyko288 | C 003 | C 003 | c2 |  |  | Tsumeb | C Namibia | Namibia | S Africa | Village dog |
| Boyko289 | C 007 | C 007 | c1 | vilC1a/vilC1b | GQ375206/GQ375207 | Armanti | Luxor | Egypt | N Africa | Village dog |
| Boyko290 | C 007 | C 007 | c1 |  |  | Ezba | Luxor | Egypt | N Africa | Village dog |
| Boyko291 | C 007 | C 007 | c1 |  |  | Comb | Luxor | Egypt | N Africa | Village dog |
| Boyko292 | C 007 | C 007 | c1 |  |  | ACE (Animal Care in Egypt) | Luxor | Egypt | N Africa | Village dog |
| Boyko293 | C 007 | C 007 | c1 |  |  | ACE (Animal Care in Egypt) | Luxor | Egypt | N Africa | Village dog |
| Boyko294 | C 008 | C 008 | c1 | vilC5 | GQ375212 | Oshivelo | N Namibia | Namibia | S Africa | Village dog |
| Boyko295 | C 008 | C 008 | c1 |  |  | Okanbjengedhi | N Namibia | Namibia | S Africa | Village dog |
| Boyko296 | C 010 | C 010 | c1 | vilC4 | GQ375211 | Jazeera | Luxor | Egypt | N Africa | Village dog |
| Boyko326 | C 021 | C 021 | c1 | vilC6 | GQ375213 | Cham-Cham | N Namibia | Namibia | S Africa | Village dog |

Table S5. Detailed structure and diversity information for the African samples from Boyko *et al.* (2009), in comparison with other regions; see Table 1 for explanations. * Unique haplotypes for Boyko *et al.* samples are assigned by excluding 58 African samples of the present study.

| Population | NABC(DEF) | nA(%) | nB(%) | nC(%) | nHT | nHTuq(%) | nHTrsp56 | HTdiv(SD) | nsubHG | %UT | %UTd |
| --- | --- | --- | --- | --- | --- | --- | --- | --- | --- | --- | --- |
| SwAsia | 336(9) | 200(58.0) | 114(33.0) | 22(6.4) | 49 | 17(34.7) | 20.59 | 0.893(0.010) | 5 | 77.7 | 94.5 |
| PersianPlateau | 164(5) | 106(62.7) | 46(27.2) | 12(7.1) | 29 | 8(27.6) | 18.25 | 0.900(0.011) | 5 | 80.5 | 92.3 |
| Anatolia | 107(4) | 64(57.7) | 35(31.5) | 8(7.2) | 28 | 5(17.9) | 18.84 | 0.881(0.019) | 4 | 76.6 | 95.5 |
| FertileCrescent | 57(0) | 30(52.6) | 26(45.6) | 1(1.7) | 13 | 2(15.4) | 12.93 | 0.868(0.024) | 4 | 71.9 | 98.2 |
| FCbelt | 92(2) | 43(45.7) | 40(42.5) | 9(9.6) | 29 | 5(17.2) | 22.25 | 0.885(0.026) | 5 | 77.7 | 93.6 |
| FCxtd | 149(2) | 73(48.3) | 66(43.7) | 10(6.6) | 33 | 7(21.2) | 20.28 | 0.885(0.019) | 5 | 75.5 | 95.4 |
| SwAexclFCxtd | 187(7) | 127(65.5) | 48(24.7) | 12(6.2) | 36 | 10(27.8) | 19.92 | 0.887(0.014) | 5 | 79.4 | 93.8 |
| SwAexclSight | 281(8) | 181(62.6) | 79(27.3) | 21(7.3) | 41 | 13(31.7) | 20.24 | 0.907(0.009) | 5 | 78.9 | 94.5 |
| SwAsianSight | 55(1) | 19(33.9) | 35(62.5) | 1(1.8) | 14 | 4(28.6) | 14 | 0.643(0.071) | 4 | 71.4 | 94.6 |
| Europe | 433(27) | 302(65.6) | 95(20.6) | 36(7.8) | 51 | 19(37.2) | 20.92 | 0.927(0.005) | 4 | 76.7 | 92.6 |
| NorthContEur | 141(1) | 90(63.4) | 30(21.1) | 21(14.8) | 22 | 3(13.6) | 15.57 | 0.916(0.007) | 4 | 91.5 | 97.9 |
| SouthEurope | 111(6) | 82(70.1) | 25(21.4) | 4(3.4) | 31 | 9(29.0) | 21.57 | 0.921(0.012) | 4 | 69.2 | 90.6 |
| Africa | 57(1) | 48(82.8) | 7(12.1) | 2(3.4) | 23 | 6(26.1) | 22.61 | 0.939(0.014) | 4 | 65.5 | 93.1 |
| IndianSubcont | 62(0) | 49(79.0) | 5(8.1) | 8(12.9) | 23 | 6(26.1) | 21.96 | 0.930(0.016) | 5 | 54.8 | 80.6 |
| Siberia | 60(2) | 39(62.9) | 13(21.0) | 8(12.9) | 22 | 8(36.4) | 21.39 | 0.947(0.013) | 4 | 50 | 72.6 |
| Japan | 118(3) | 76(62.8) | 24(19.8) | 18(14.9) | 28 | 8(28.6) | 20.94 | 0.941(0.007) | 7 | 58.7 | 82.6 |
| NorthernChina | 273(0) | 200(73.3) | 52(19.0) | 21(7.7) | 44 | 16(36.4) | 21.37 | 0.924(0.007) | 7 | 71.4 | 82.8 |
| ASY | 339(2) | 273(80.1) | 48(14.1) | 18(5.3) | 88 | 54(61.4) | 30.07 | 0.959(0.004) | 10 | 40.5 | 52.5 |
| EastAsia | 855(12) | 658(75.9) | 133(15.3) | 64(7.4) | 131 | 101(77.1) | 28.9 | 0.952(0.003) | 10 | 54.9 | 68.6 |
| WestEurasia | 769(36) | 502(62.4) | 209(26.0) | 58(7.2) | 74 | 37(50.0) | 21.81 | 0.916(0.005) | 5 | 77.1 | 93.4 |
| Boyko *et al.* | 309(0) | 229(74.1) | 59(19.1) | 21(6.8) | 42 | 14(33.3)* | 22.1 | 0.926(0.007) | 7 | 74.4 | 92.9 |

Table S6: Detailed information on sharing of haplotypes for SwAsia, Europe, and Siberia.

| Region | N | nHT | Private HTs | HTs shared exclusively with Siberia | HTs shared exclusively with SwAsia | HTs shared with both Siberia and SwAsia |
| --- | --- | --- | --- | --- | --- | --- |
| Europe | 460 | 51 | 23 | 2 | 16 | 10 |
| SwAsia | 345 | 49 | 22 | 1 | - | - |
| Siberia | 62 | 22 | 9 | - | - | - |

Table S7: Detailed information on sharing of haplotypes among sighthound groups. UTs included are shown in grey shade. The groups contained breeds/ecotypes/local populations as follows:

East SwAsia: Afghan Hound, Kalagh-Tazi, Bakhmul, Taigan

West SwAsia: Persian Greyhound, Saluki, Tazi

North Africa: Azawakh, Sloughi

South Europe: Galgo Espanol, Piccolo Levriero Italiano

East Europe: Borzoi, Hortaya borzaya, Stepnaya, Chart Polski, Magyar Agar

British Isles: Greyhound, Irish wolfhound, Scottish deerhound, Whippet

| HT | East SwAsia | West SwAsia | North Africa | South Europe | East Europe | British Isles |
| --- | --- | --- | --- | --- | --- | --- |
| A 004 | - | - | 2 | - | - | - |
| A 005 | - | - | 1 | - | - | - |
| A 009 | - | 2 | - | - | - | - |
| A 010 | - | - | 2 | - | - | - |
| A 011 | 3 | 2 | 3 | 4 | 1 | 6 |
| A 012 | 5 | - | - | - | - | - |
| A 015 | - | 1 | - | - | - | - |
| A 016 | - | - | - | - | - | 1 |
| A 017 | - | - | - | - | - | 2 |
| A 018 | 1 | - | - | - | - | - |
| A 020 | - | - | - | - | 4 | - |
| A 022 | - | - | 1 | - | 2 | 4 |
| A 023 | - | - | - | - | 1 | - |
| A 024 | - | - | - | - | - | 3 |
| A 026 | - | 2 | - | - | - | - |
| A 027 | - | 1 | - | - | - | - |
| A 033 | - | - | 1 | 1 | - | - |
| A 034 | - | - | - | 2 | - | - |
| A 043 | - | 1 | - | - | - | - |
| A 127 | - | - | 2 | - | - | - |
| A 134 | - | 1 | - | - | - | - |
| A 168 | - | - | - | - | 1 | - |
| A 170 | - | - | - | - | 1 | - |
| B 001 | 11 | 22 | 1 | 3 | 2 | - |
| B 004 | - | 1 | - | - | - | - |
| B 015 | - | 1 | - | - | - | - |
| C 001 | - | 1 | - | - | - | - |
| C 003 | - | - | - | - | 1 | - |
| D 006 | - | - | - | 5 | - | - |
| D 007 | - | - | 1 | - | - | - |
| D 010 | - | 1 | - | - | - | - |
| Total | 20 | 36 | 14 | 15 | 13 | 16 |
