# Supplementary material for: Comprehensive study of mtDNA among Southwest Asian dogs contradicts independent domestication of wolf, but implies dog–wolf hybridization
Source: Ecol Evol. 2011 Nov;1(3):373–85. doi: 10.1002/ece3.35 (PMC3287314; doi:10.1002/ece3.35)

Figure S1: MS networks for the African samples reported by Boyko et al. (2009). See Figure 3 for explanations.

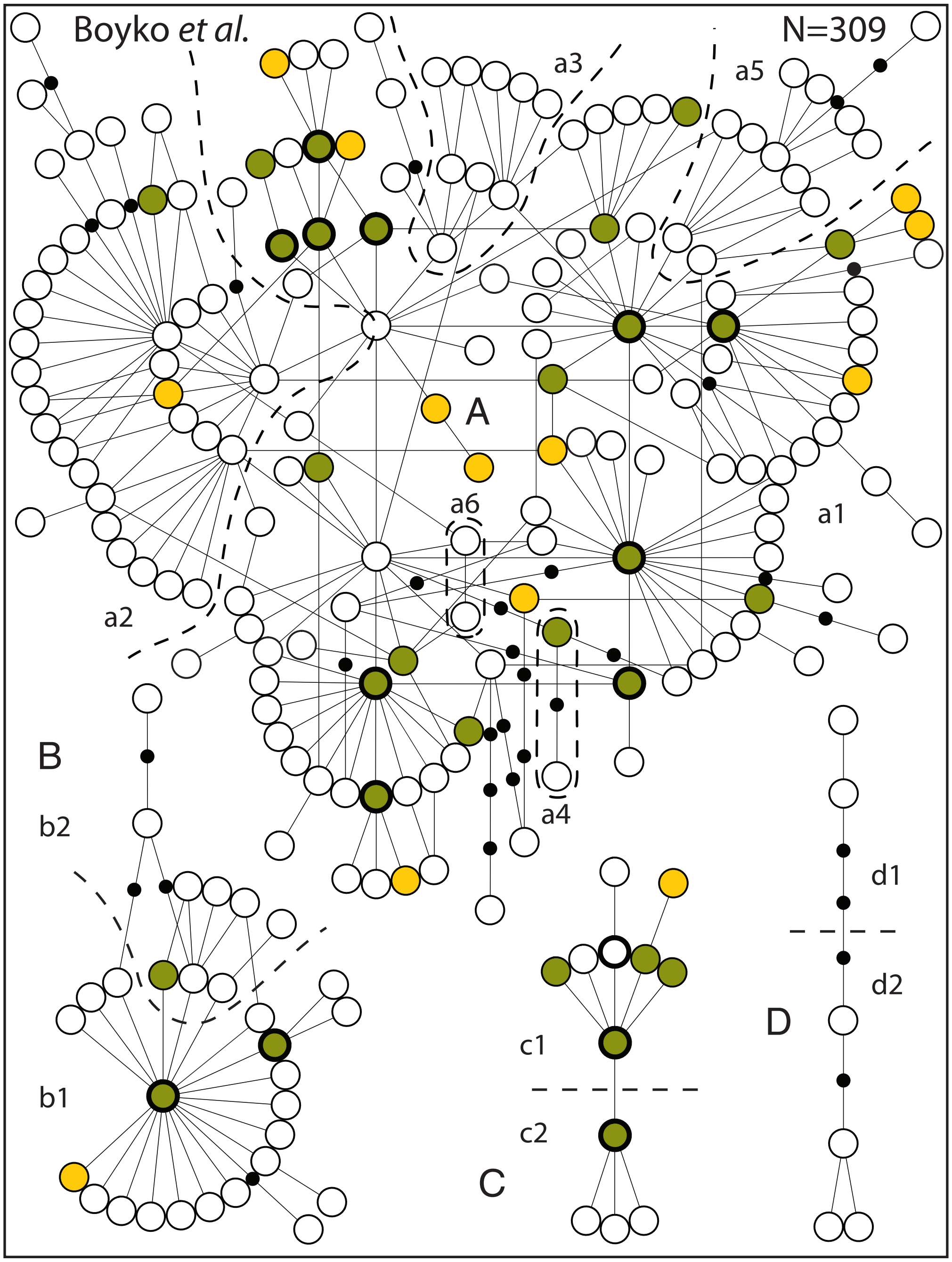

Supplement: Supplementary file 3 [file ece30001-0373-SD3.pdf]
