# Supplementary material for: Comprehensive study of mtDNA among Southwest Asian dogs contradicts independent domestication of wolf, but implies dog–wolf hybridization
Source: Ecol Evol. 2011 Nov;1(3):373–85. doi: 10.1002/ece3.35 (PMC3287314; doi:10.1002/ece3.35)

Figure S2: Topology of the MS networks and the sub-haplogroups, with haplotype numbers shown. Haplotypes with grey shade were left unassigned to sub-haplogroups, based on the diagnostic point mutations (Pang *et al.* 2009). See Figure 3 for more explanations.

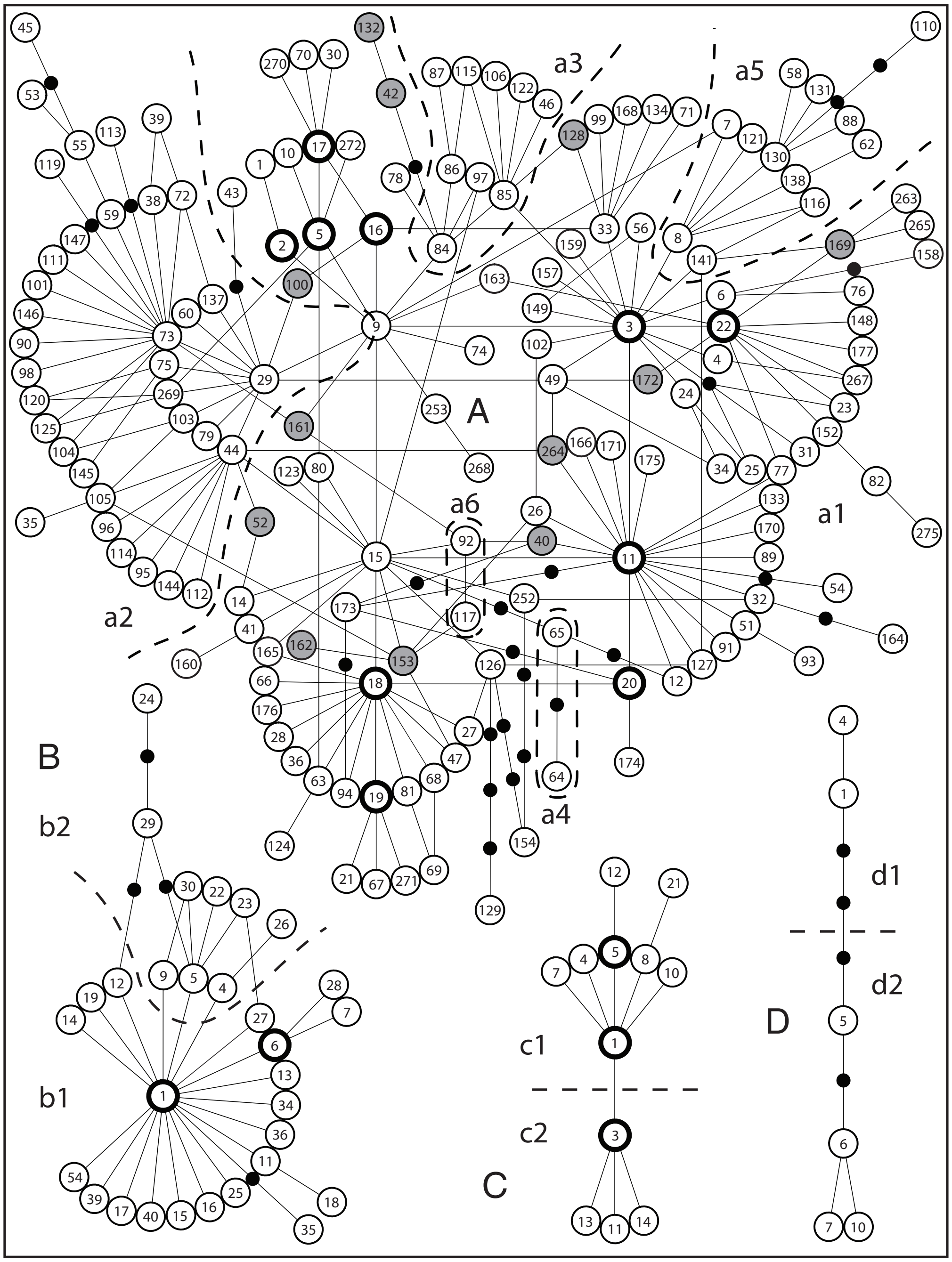

Supplement: Supplementary file 4 [file ece30001-0373-SD4.pdf]
